# Global distribution, diversity, and ecological niche of Picozoa, a widespread and enigmatic marine protist lineage

**DOI:** 10.1186/s40168-024-01874-1

**Published:** 2024-09-04

**Authors:** Paula Huber, Daniele De Angelis, Hugo Sarmento, Sebastian Metz, Caterina R. Giner, Colomban De Vargas, Luigi Maiorano, Ramon Massana, Ramiro Logares

**Affiliations:** 1 Departamento de Hidrobiología, Universidade Federal de São Carlos, São Carlos, Brazil; 2 Dipartimento Di Biologia E Biotecnologie “Charles Darwin”, Università Di Roma La Sapienza, Rome, Italy; 3 Department of Archaeology, University of York, York, UK; 4 Institut de Ciències del Mar (ICM), CSIC, Barcelona, Catalonia Spain; 5 Sorbonne Universités, CNRS, Station Biologique de Roscoff, Roscoff, France; 6 Research Federation for the Study of Global Ocean Systems Ecology and Evolution, Paris, France

**Keywords:** Picozoa, Protist, Marine picoeukaryotic, Heterotrophic flagellates, Molecular diversity, Species distribution modeling, Ecological niche, Phylogenetic overdispersion

## Abstract

**Background:**

The backbone of the eukaryotic tree of life contains taxa only found in molecular surveys, of which we still have a limited understanding. Such is the case of Picozoa, an enigmatic lineage of heterotrophic picoeukaryotes within the supergroup Archaeplastida, which has emerged as a significant component of marine microbial planktonic communities. To enhance our understanding of the diversity, distribution, and ecology of Picozoa, we conduct a comprehensive assessment at different levels, from assemblages to taxa, employing phylogenetic analysis, species distribution modeling, and ecological niche characterization.

**Results:**

Picozoa was among the ten most abundant eukaryotic groups, found almost exclusively in marine environments. The phylum was represented by 179 Picozoa’s OTU (pOTUs) placed in five phylogenetic clades. Picozoa community structure had a clear latitudinal pattern, with polar assemblages tending to cluster separately from non-polar ones. Based on the abundance and occupancy pattern, the pOTUs were classified into four categories: Low-abundant, Widespread, Polar, and Non-polar. We calculated the ecological niche of each of these categories. Notably, pOTUs sharing similar ecological niches were not closely related species, indicating a phylogenetic overdispersion in Picozoa communities. This could be attributed to competitive exclusion and the strong influence of the seasonal amplitude of variations in environmental factors, such as temperature, shaping physiological and ecological traits.

**Conclusions:**

Overall, this work advances our understanding of uncharted protists’ evolutionary dynamics and ecological strategies. Our results highlight the importance of understanding the species-level ecology of marine heteroflagellates like Picozoa. The observed phylogenetic overdispersion challenges the concept of phylogenetic niche conservatism in protist communities, suggesting that closely related species do not necessarily share similar ecological niches.

Video Abstract

**Supplementary Information:**

The online version contains supplementary material available at 10.1186/s40168-024-01874-1.

## Introduction

Marine microbes play a fundamental role in shaping the Earth’s ecosystem, governing global biogeochemical cycles, and facilitating the transfer of matter and energy to higher trophic levels [1–3]. Among them, protists are key components of the marine microbiome and fulfill a vast array of ecological roles due to their wide variety of physiological capacities [4–6]. Within the expansive and complex world of marine protists, a group of small heterotrophic picoeukaryotes known as Picozoa has emerged as a fascinating and enigmatic lineage.

Picozoa were described for the first time in 2007 as a unique photosynthetic protist lineage, called Picobiliphytes [7]. The assignment of this nutrition strategy was based on a distinct orange autofluorescence (apparently phycobilin pigments) emitted by the cells when observed under epifluorescence microscopy [7]. However, subsequent studies failed to find phycobilin in natural populations, concluding that Picozoa were not autotrophs but rather phagotrophs and that their orange fluorescence could represent ingested picocyanobacteria [8]. The question of whether or not Picozoa were autotrophs seemed to be resolved by the morphological characterizations of a single strain of Picozoa (*Picomonas judraskeda*) [9]. This strain was characterized as a biflagellate, with cells consisting of two hemi-spheres with structural features that have not been observed before in any other eukaryote. The anterior part contains the typical eukaryotic organelles, whereas the posterior part contains numerous vesicles and vacuoles and the feeding apparatus. These two parts are separated by a vacuolar cisterna of unknown function [9]. At present, it remains unknown if this morphological description is universally applicable to all members of this group. The absence of chloroplast and the feeding apparatus suggested that this species was adapted to exploit small particles as a food source. Therefore, Picobiliphytes was renamed as “Picozoa” highlighting their heterotrophic lifestyle [9, 10, 11, 12]. In agreement, the genome sequencing of three single cells showed no evidence of plastid DNA or plastid-targeted proteins [2], although this was based on incomplete genomes. Furthermore, viral and bacterial genes were found together with these genomes, suggesting that Picozoa may feed on these organisms [12]. A recent single-cell genomics study based on a wide range of cells has definitely refuted the presence of chloroplasts in Picozoa [13].

The phylogenetic position of Picozoa has also been a subject of uncertainty. For several years, multiple studies have characterized the group as an orphan basal lineage, distinct from any established eukaryotic cluster [7, 12, 14–16]. However, a recent phylogenomic study revealed that Picozoa belongs to the Archaeplastida supergroup. Archaeplastida comprises diverse photosynthetic lineages from primary endosymbiosis (green algae, red algae, and glaucophytes), where a eukaryotic host cell engulfed a cyanobacterium, giving rise to their plastids [13]. In addition to these photosynthetic lineages, Archaeplastida also includes heterotrophic groups like Rhodelphis [17] and, more recently, Picozoa, both basal to red algae [13]. The coexistence of a few heterotrophic lineages alongside photosynthetic lineages highlights the complexity of this supergroup [18]. Indeed, rhodelphids lost their plastid genome over time, but the plastid organelle remains [17]. So, these organisms are obligate phagotrophs preserving cryptic non-photosynthetic plastids [17]. Picozoa, however, lacks a plastid and shows no evidence of an early cryptic endosymbiosis with cyanobacteria [13]. This unique scenario raises the possibility that this group could be the first example of complete plastid loss in a free-living taxon. Alternatively, it may suggest that red algae and rhodelphids obtained their plastids independently from other archaeplastids [13].

Over the few last years, the presence of Picozoa in molecular surveys has exhibited a sustained increase across the global ocean, from temperate and tropical waters [7, 15, 19–21] to polar regions [22–25]. Recently, an amplicon dataset retrieved from samples from tropical and subtropical oceanic regions has revealed that Picozoa constitute more than 10% of the relative abundance of heterotrophic flagellates in surface samples, positioning them as the second most dominant HFs group after MAST-3 [21]. These findings highlight the central role of Picozoa in marine microbial planktonic communities. However, key aspects of their diversity, distribution, and ecological significance remain poorly understood.

The ecological niche theory may not only shed light on the significance of Picozoa in marine microbial planktonic communities but also provide a framework for a deeper understanding of their ecological implications. The traditional niche-based perspective states that selection, including environmental filtering and species interactions, plays a pivotal role in shaping community structure [26]. This process drives species into specific niches based on their ecological requirements and interactions with other species, which are then balanced by stochastic processes (birth, death, colonization, immigration, speciation, and probabilistic dispersal) [26, 27]. Together, these deterministic and stochastic forces shape community assembly [28, 29]. In protists, selection and dispersal limitation are often considered the main ecological drivers of species distributions [30–36]. However, these statements are valid for communities in general, and it is expected that different taxonomic groups or lineages are structured by different processes [37]. Phylogenetic niche conservatism (PNC) is an eco-evolutionary process that leads closely related taxa to share similar ecological niches due to their shared evolutionary history [38]. This implies that they are often filtered into the same habitats and tend to co-occur within these environments [39]. The opposite scenario to PNC is competitive exclusion when closely related species require the same resource and co-exclude themselves. Under this scenario, high phylogenetic overdispersion is expected, as closely related species avoid each other due to high niche overlap which leads to high resource competition [40]. Furthermore, the niche convergence processes can result in species sharing similar ecological niches without being necessarily evolutionary closely related [41].

**Fig. 1:**
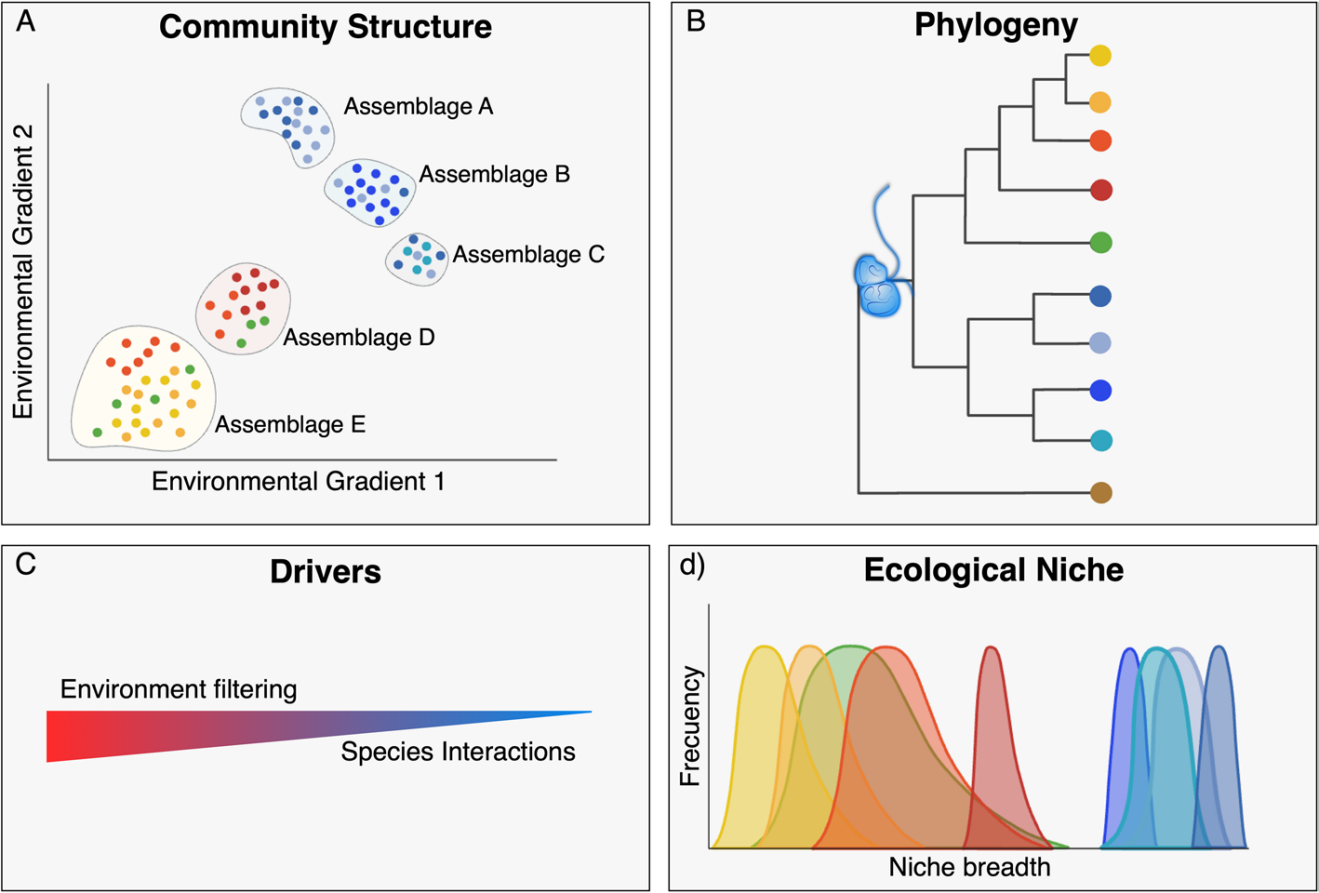
Working hypotheses on the distribution and ecological niche of Picozoa. **A** Picozoa communities are latitudinally structured, with polar (tones of blue) assemblages clustering separately from non-polar ones (other colors). **B** Selection (or environmental filtering) is expected to play a larger role in this spatial distribution than species interactions, with temperature being one of the main drivers shaping the assemblage structure. **C**, **D** Consequently, Picozoa taxa sharing similar distribution patterns are likely to be phylogenetically related, indicating phylogenetic niche conservatism

In this study, we aim to unveil the ecology of Picozoa through a global-scale phylogenetic and niche characterization. As Picozoa is a widely distributed and abundant picoeukaryote group in the global ocean, we hypothesize that the different species composing the group display a latitudinal distribution mainly influenced by environmental factors. As a consequence, phylogenetically related taxa are expected to co-occur, sharing and occupying similar ecological niches (Fig. 1). To test our hypotheses, we analyze an extensive dataset of 18S rRNA gene V4 sequences to (1) determine spatial distribution patterns at different levels, from assemblages to OTUs, (2) place the detected OTUs into a proper phylogenetic context, and (3) study the ecological niche dynamics at the OTU level.

## Material and methods

### EukBank 18S rRNA—V4 region database

The EukBank database compiles eDNA surveys from 12,570 georeferenced samples that used amplicon high-throughput sequencing methods (Illumina MiSeq and Roche 454) to target the hypervariable V4 region of the 18S rRNA gene [42]. This database comprised samples from both continental and marine environments, targeting different microbial size fraction: pico-size (0.2–5 µm), nano-size (5–20 µm), and micro-size (> 20 µm) (Supplementary Table S1).

Raw sequences were obtained from the EMBL/EBI-ENA EukBank umbrella project. When applicable, reads were trimmed with Cutadapt [43] to extract fragments covered by the primer sets TAReuk454FWD1 and TAReukREV3 from the V4 region or the 18S rRNA gene [44]. Identical sequences were merged with VSEARCH [45] and clustered with Swarm [46]. Subsequently, chimera detection and removal were conducted using the *–uchime_denovo* function in VSEARCH [45]. The final set of operational taxonomic units (OTUs) was obtained based on occurrence patterns, utilizing a modified version of the Lulu algorithm [47]. Taxonomic classification of the OTUs was performed using the curated EukRibo database version 1.0 [48], employing the global pairwise alignment approach (*–usearch_global* from VSEARCH). This taxonomically informed OTU table was generated by the UniEuk consortium [42]. Only samples with more than 10,000 reads were considered and rarefied using the *rrarefy* function from “Vegan” package [49] in the R environment [50]. The 223 OTUs that were initially affiliated with Picozoa (pOTUs) were corroborated by their phylogenetic placement in an 18S rRNA phylogenetic reference tree (see next section) and manually checked for chimeras. We kept 179 pOTUs together with their relative abundance in all samples for further analysis.

### Phylogenetic analysis

The Picozoa reference tree (RT) was constructed using 18S rRNA sequences from the PR2 database (release v. 5.0.1 [51]). Sequences named Picozoa were downloaded (289 sequences longer than 800 bp). A preliminary tree indicated the presence of long branches within clades and at the base of the tree. A manual inspection of these long branches showed that many of them were chimeras (20 sequences), had erroneous bases at the start or end (25 sequences), or were misassigned. Subsequent analysis indicated that most partial sequences did not modify the topology of the tree, so we finally kept 50 almost complete sequences for the reference tree (47 longer than 1600 bp and 3 between 1000 and 1600 bp). Twelve Cryptophyte sequences retrieved from the PR2 database were used as outgroup. These sequences were aligned using MAFFT v.7 software [52] with the strategy G-INS-i, and the RT was constructed using the maximum likelihood method in RAxML v.8.2.12 [53] with the GTRCATI model considering 1000 trees for topology and 1000 trees for bootstrapping. Clades names of Picozoa were based on this tree.

To infer the phylogenetic positions of the pOTUs, the amplicon short sequences were placed into the RT. Briefly, the pOTUs sequences were added to the reference alignment using MAFFT with the *–add* and *–keeplength* parameters. Then, a maximum likelihood phylogenetic tree was constructed using RAxML with the GTRCATI considering 1000 replicates for topology and bootstrapping. All pOTUs were clearly placed within the lineages described in the reference tree.

### Community structure and diversity

To investigate the community structure and diversity of Picozoa across the global ocean, we focused only on DNA samples (not RNA) sequenced by Illumina technology, belonging to marine water environment, from the pico-size fraction (i.e., collected using filters with pore sizes ranging from 0.2 to 5 µm, or that have the information in sample description). For methodological consistency, a single sample per location was considered in the dataset, excluding any times series or replicate samples. This refined dataset comprised 2366 samples collected from diverse locations, spanning from polar regions to the equator, and encompassing the sunlit (depth layer between 0 and 200 m, or as per sample description, comprising the surface and epipelagic) and dark ocean (depth layer deeper than 200 m, or as per sample description, comprising the meso- and bathypelagic).

To explore the difference in community composition between sunlit and dark ocean zones, we first conducted a PERMANOVA test [54]. Then, we calculated Shannon–Weaver diversity (H′) and CHAO-1 richness indices from rarefied OTU tables (10,000 reads), and evaluated significant differences (*P* < 0.01) among sunlit and dark ocean zones using a *t*-Student test.

To further analyze the structure of Picozoa communities in the sunlit ocean (1669 samples), we conducted a non-metric multidimensional scaling ordination plot (NMDS) based on the Bray–Curtis dissimilarity on relative abundance pOTU tables using the “Vegan” R package [49]. Then, we conducted a PERMANOVA analysis to test for significant differences in the assemblage structure among latitude ranks.

### Picozoa biogeography and ecological niche

To study the Picozoa biogeography, we used two different approaches. First, we focused on field observations at the pOTU level in the sunlit ocean to gather comprehensive data on Picozoa abundance and distribution. Then, we employed Species Distribution Models (SDMs) to predict the potential distribution of each abundant pOTU (53 pOTUs with a total abundance > 150 reads and occurrence > 50; see results) at the global scale. Our analysis categorized Picozoa into four distinct groups. Finally, we investigate the ecological niche of each abundant pOTU, to determine their abiotic environmental preferences.

#### Field observations

Picozoa OTUs were classified into four categories based on abundance, occupancy, and statistical association to one or more oceans (*IndVal.g* function in the “Indicspecies” R package [55]). We considered a pOTU to be significantly associated with one ocean when the IndVal.g association value was 1 and *p*-value < 0.05 (9999 permutations (55)). These categories included the following: (1) Low abundance (LA), (2) Widespread (W), (3) Polar (P), and (4) Non-polar (NP).

#### Species distribution modeling

##### Selection of environmental predictors

To describe the predicted biogeographical patterns of Picozoa at the global scale, we selected a set of mean yearly climatologies for seven environmental variables that better describe the biogeographical patterns of protists at the global scale (e.g., [21]). These predictors were obtained from the World Ocean Atlas 2018 (https://www.ncei.noaa.gov/products/world-ocean-atlas) and integrated into the standard 1 × 1 global grid. They included: mixed-layer depth and multi-depth (0–5500 m) fields for temperature, salinity, oxygen, conductivity, phosphates, and nitrates concentrations. Given the high spatial correlation between phosphates and nitrates concentrations, and to reduce the total number of predictors in models, we computed the excess of nitrates over phosphates (N*) based on the Redfield ratio [NO_3−_] − 16[PO_4_^3−^] [56]. Higher values of this variable indicated areas with a clear excess of nitrates over phosphates. Finally, we ensured that none of the variables included in the models had a variance inflation factor higher than three. Multi-depth climatologies were averaged across distinct depth layers to represent mean yearly conditions for sunlit (0–200 m), mesopelagic (> 200–1000 m), and bathypelagic (> 1000 m) ocean depth zones, respectively.

##### Species Distribution Models configuration and habitat suitability projections

Species Distribution Models (SDMs) were used to predict the potential distribution of each abundant pOTU at the global scale based on a set of abiotic environmental conditions. SDMs are a widely used technique that contrasts environmental conditions between species occurrence and background data to estimate habitat suitability and predict species distribution across geographical space [57]. pOTU occurrences were compiled by discretizing read counts into presence/absence data, retaining only one observation for each cluster of reads collected within the same ocean depth zone. To minimize bias in the sampling effort, we employed a target-group approach [58], restricting the selection of background data to the spatial boundaries of our sampling. Specifically, locations where a pOTU was found were matched with depth-specific conditions and used as presence data. Meanwhile, depth-specific conditions for all other sampling stations, where a pOTU was not found, served as background data. This approach implied dealing with different presence-to-absence ratios in the training data (mean = 0.17, range 0.02–0.83) for each modeled pOTU, reflecting the diverse relative abundance of pOTU in our global dataset [59, 60]. We included an average of 202.8 occurrences per pOTU in SDMs (range, 24–725).

The SDMs were fitted using an ensemble of 4 algorithms: Generalized Linear Models (GLM), Random Forest (RF), Artificial Neural Network (ANN), and Boosted Regression Trees (BRT) [61]. The models were calibrated using fivefold cross-validation to internally assess their performance based on the area under the receiving operator characteristic curve (AUC). For each pOTU, models with poor performance (AUC < 0.7) were discarded from the final ensemble model, which was calculated as the average of predictions across all successful algorithms [61]. Only 17 models were discarded, while the retained models exhibited an average performance across all pOTUs at 0.87 (range, 0.72–0.99). The calibrated SDMs were then projected onto the yearly sunlit conditions to generate global maps of the Habitat Suitability Index (HSI) for each pOTU. The HSI index helps to predict potential changes in species distribution across different habitats. It ranges from 0 to 1, where 1 represents the maximum probability of finding a species in a given environment, indicating the highest suitability for the species. This analysis was conducted using the “h2o” [62] and “raster” [63] R packages.

#### Ecological niche and niche overlap

The ecological niche was estimated for all abundant pOTUs by the canonical Outlying Mean Index analysis (OMI) [64, 65]. OMI is an ordination technique that had been found to characterize the ecological niche more accurately than SDMs [66]. The OMI technique measures marginality, which is the distance between a species’ average environmental preferences and the overall conditions available in the sampled area. This analysis was performed on the ecological space defined by a principal component analysis (PCA) applied to the table containing abiotic environmental variables. The analysis returns the linear combination of habitat variables that maximizes the mean marginality of the species. Consequently, each species is positioned in the gridded, multivariate ecological space based on the deviation of its niche from that of a hypothetical species uniformly distributed across available environmental conditions [64, 65]. As we expected temperature to strongly structure environmental conditions, we used a slight modification of the classical OMI, called canonical OMI (CANOMI), which is specifically indicated in such situations ( “adehabitatHR” R package [65, 67]). We performed the CANOMI analysis using information on the number of reads per pOTU counted at each sampling station. We represented available conditions by extracting, for each sampling station, the same variables included in SDMs for the sunlit ocean.

To quantify niche overlap between different pOTUs, we first used a kernel distribution (*kernelUD* function in “adehabitatHS” R package [68]) to determine the “smoothed” density of pOTU reads counted in each grid of the ecological space from the CANOMI analysis [66]. Then, we employed the Schoener’s D metric (*calc.niche.overlap* functions in “ENMeval” R package [69]), to compute the niche overlap between each pair of pOTU. Schoener’s D metric spans from 0, indicating no overlap, to 1, representing complete overlap [66].

### Phylogenetic community structure and phylogenetic niche conservatism

The phylogenetic community structure was assessed using the mean nearest taxon distance (MNTD) index, which calculates the mean phylogenetic distance separating each species in the community from its closest relative [70]. A low MNTD value indicates closely related species, while a high MNTD value suggests more distantly related species [70]. This analysis was conducted using the “Picante” R package [71].

The PNC was estimated to test whether the environmental preference of a given abundant pOTU was related to the phylogeny. We employed two different approaches. First, a Mantel correlogram analysis was run to assess the relationship between potential environmental traits and phylogenetic distances of pOTUs [72–76]. The potential environmental trait information for each taxon was obtained by calculating the average values of each environmental variable (the same used in SDMs and OMI analyses) for the sites in which it was observed, weighted by the relative abundance of that taxon per site. The phylogenetic distance between pOTUs was calculated from the ML 18S rRNA tree using the cophenetic function in R environment (50). The Mantel correlogram analyses were run with 999 permutations using the “Vegan” R package [49], employing 50 phylogenetic distance bins and a progressive Bonferroni correction. Then, we also explored the correlation between pOTUs’ phylogenetic distance and their niche overlap.

## Results

### Global distribution, diversity, and phylogeny of Picozoa

**Fig. 2:**
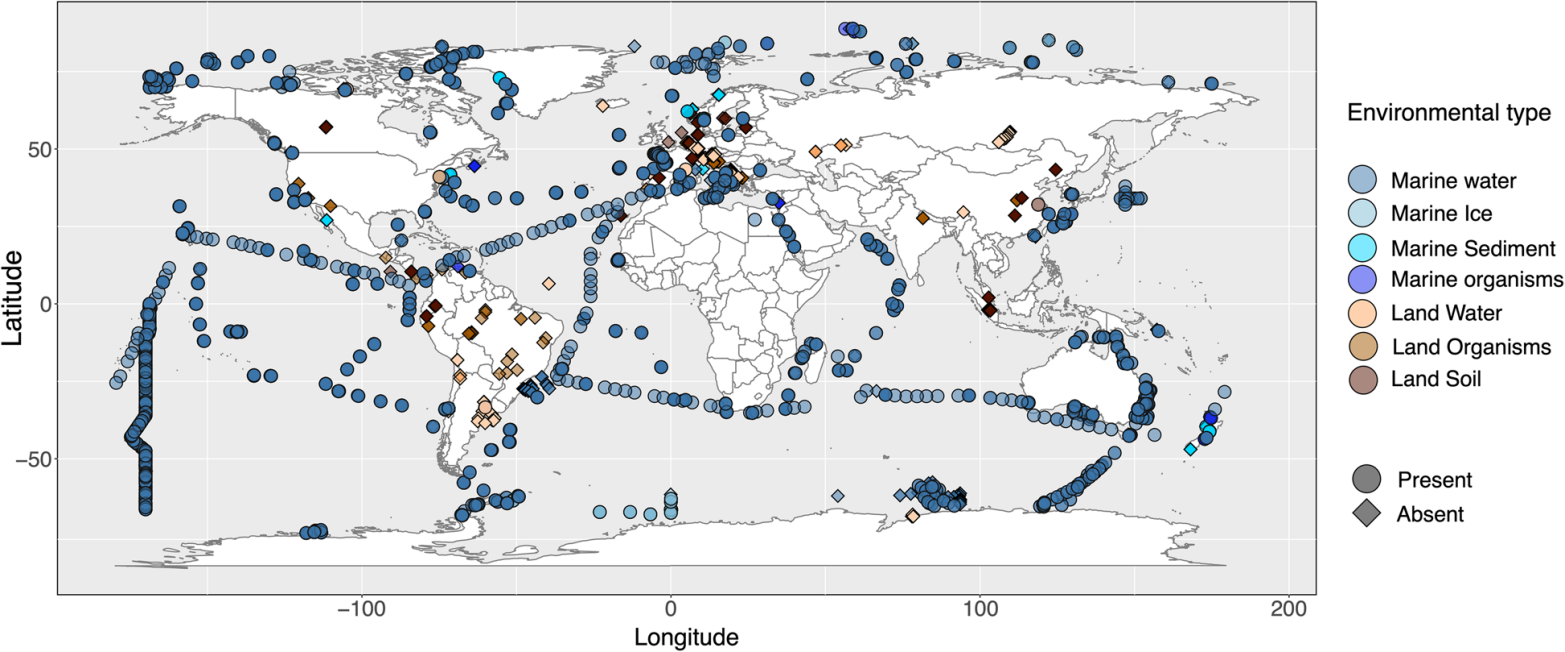
The spatial distribution of Picozoa. This figure shows the presence and absence of Picozoa sequences in different environments within the EukBank database, encompassing 12,570 georeferenced samples

In the Eukbank 18S rRNA amplicon dataset (Supplementary Table S1), Picozoa ranked among the ten most abundant supergroups (Supplementary Fig. S1), contributing to 0.5% of total eukaryotic reads, comprising 179 pOTUs. The predominant groups in terms of abundance can be observed in Supplementary Fig. S1. Notably, Picozoa occurred systematically in marine environments, being found in 85.1% of marine samples, contributing significantly, up to 37%, to the total eukaryotic abundance. Its presence was very scarce in continental environments (Fig. 2 and Supplementary Fig. S2). They were found in only 35 of 2876 continental samples, and they contributed very little to read abundance, as almost all Picozoa reads (99.9%) originated from marine water samples.

Among samples from marine water environments (*n* = 7886), Picozoa constituted more than 5% of the total eukaryotic reads in 174 samples, with the majority of these samples originating from polar regions (Supplementary Fig. S3). Picozoa reads were distributed across the pico-, nano-, and micro-sized fractions. The pico-sized fraction displayed the highest number of taxa and reads among the three fractions. There was considerable overlap between the three size fractions in terms of pOTUs, with all pOTUs from the nano- and micro-sized fractions also being present in the pico-size fractions. However, 116 out of 179 pOTUs were exclusive to the pico-size fraction (Supplementary Table S2).

Based on these results, we focused exclusively on pico-size marine samples (2394 samples, after excluding time series and replicates). PERMANOVA analysis indicated substantial assemblage differences between sunlit and dark ocean zones (sum of SS = 43.44; p.adjusted = 0.001). Notably, the sunlit ocean exhibited significantly higher abundance, richness, and diversity (*t*-Student, *p* < 0.001; Supplementary Fig. S4). Also, it was observed that 54 pOTUs were exclusive to the dark ocean (Supplementary Table S2). However, these pOTUs accounted for no more than 0.03% of the total reads, and showed a low occurrence across the samples. Notably, only 2 pOTUs exhibited an occurrence exceeding 5%: pOTU65 at 6.7%, and pOTU57 at 8.8% (Supplementary Table S2).

**Fig. 3:**
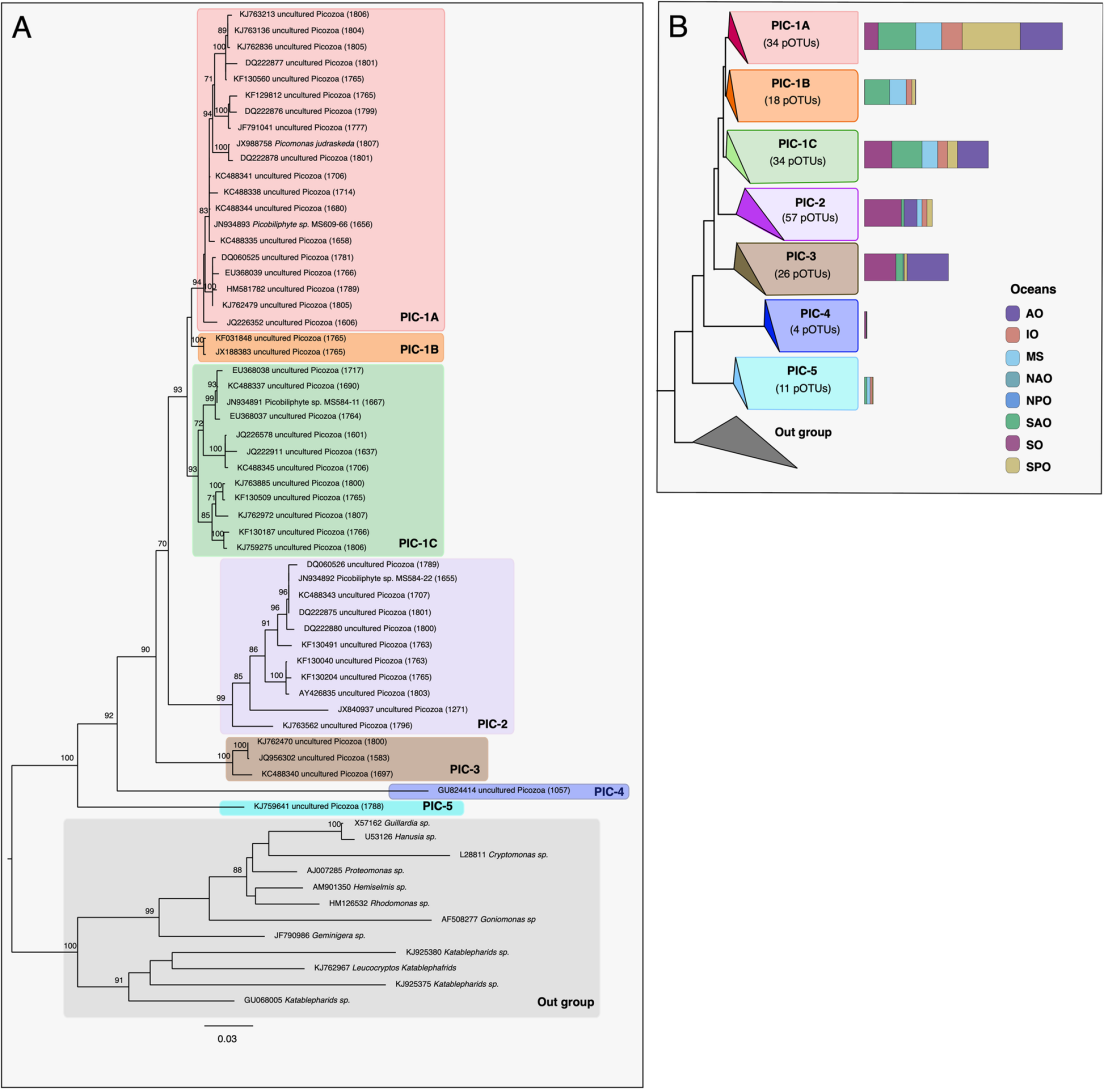
Maximum likelihood tree of Picozoa. **A** 18S rRNA reference tree constructed using 50 almost complete Picozoa sequences retrieved from the PR2 database (release v. 5.0.1). The tree was constructed in RAxML v.8.2.12 [53] with the GTRCATI model considering 1000 replicate trees for topology and 1000 trees for bootstrapping. Clades of Picozoa were based on this tree. **B** Phylogenetic representation of Picozoa and abundance distribution of clades across different oceans (AO Arctic Ocean, IO Indian Ocean, MS Mediterranean Sea, NAO North Atlantic Ocean, NPO North Pacific Ocean, SAO South Atlantic Ocean, SO Southern Ocean, SPO South Pacific Ocean) based on pOTU assignments (indicated in each clade)

Phylogenetic tree reconstruction using almost complete 18S rRNA gene reference sequences showed that the diversity of Picozoa is composed of a main lineage (PIC-1) that could be subdivided into three subclades and four additional basal groups (Fig. 3A and Supplementary Fig. S5). Several of the basal branches in previous trees were chimeras. Interestingly, all the branches of the reference tree get populated by the pOTUs from the EukBank database (Fig. 3B and Supplementary Fig. S5). The clades PIC-2 and PIC-1A exhibited the highest number of taxa (57 pOTUs and 34 pOTUs, respectively), with PIC-1A being the most abundant clade. PIC-1B (18 pOTUs) and PIC-5 (11 pOTUs) were predominantly absent in polar regions, whereas PIC-3 (26 pOTUs) and PIC-4 (6 pOTUs) showed greater abundance in the Southern Ocean (Fig. 3B). PIC-1C and PIC-1A tend to display a cosmopolitan distribution (Fig. 3B). The 54 pOTUs exclusive to the dark ocean were dispersed across different clades (Supplementary Table S2).

### Picozoa biogeography

**Fig. 4:**
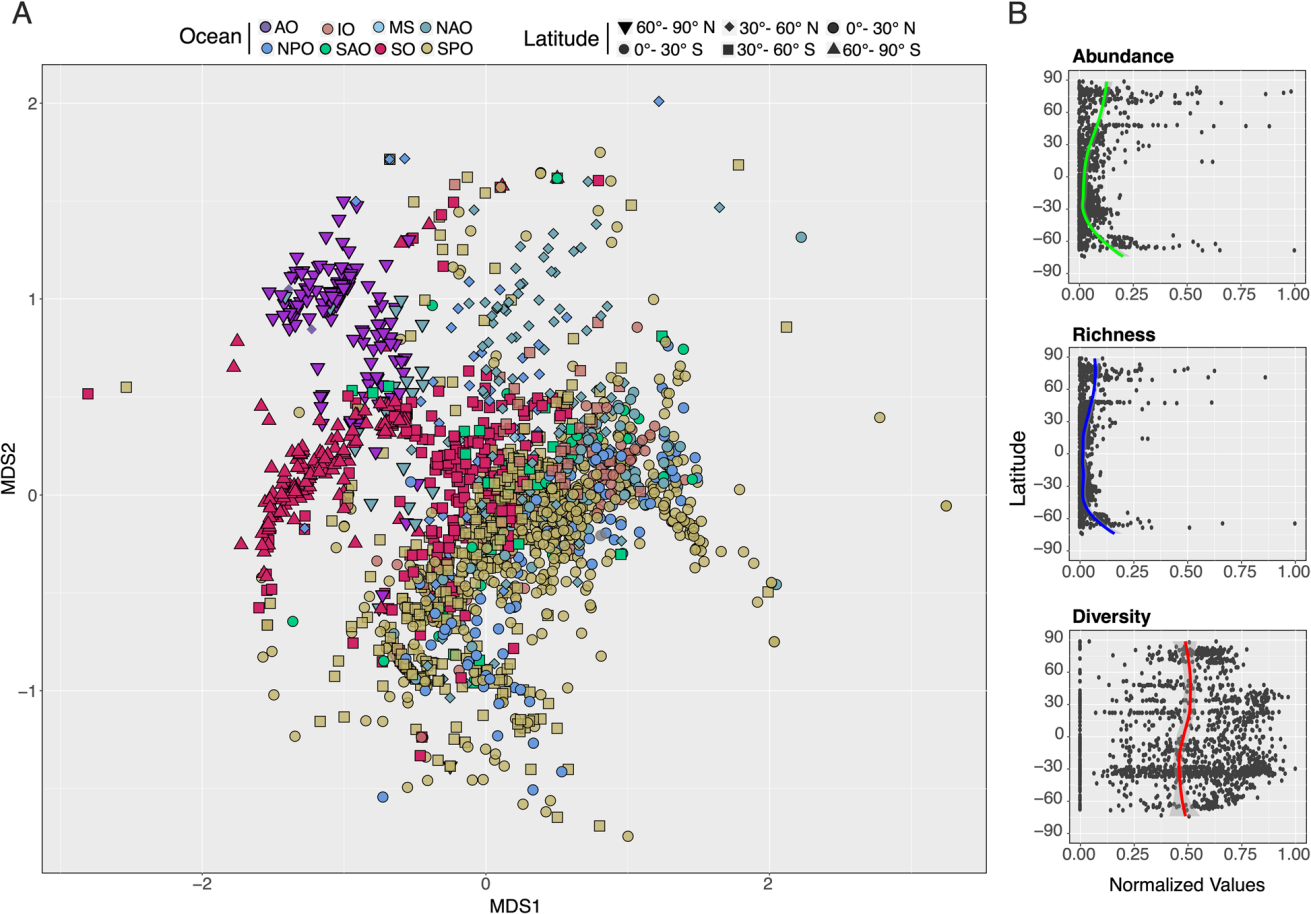
Picozoa community structure in the sunlit global ocean. **A** Sample ordination in a non-metric multidimensional scaling (NMDS) plot according to the similarity in Picozoa community structure (based on pOTUs relative abundance). Different oceans are indicated with color and latitudinal rank with symbols (AO Arctic Ocean, IO Indian Ocean, MS Mediterranean Sea, NAO North Atlantic Ocean, NPO North Pacific Ocean, SAO South Atlantic Ocean, SO Southern Ocean, SPO South Pacific Ocean). **B** Latitudinal variation of the Abundance, Richness, and Shannon–Weaver diversity index H′ of local Picozoa communities

Community structure in the sunlit ocean (118 pOTUs in 1669 samples) showed a clear latitudinal pattern, with polar communities (60–90° N and 60–90° S) tending to cluster separately from non-polar communities (Fig. 4A). PERMANOVA analysis indicated substantial assemblage structure between latitudes ranks (p.adjusted < 0.05; Supplementary Table S3). The maximum abundance of pOTUs was detected in polar regions, with slightly lower richness in tropical and subtropical regions. In contrast, the diversity did not show a clear latitudinal pattern (Fig. 4B).

**Fig. 5:**
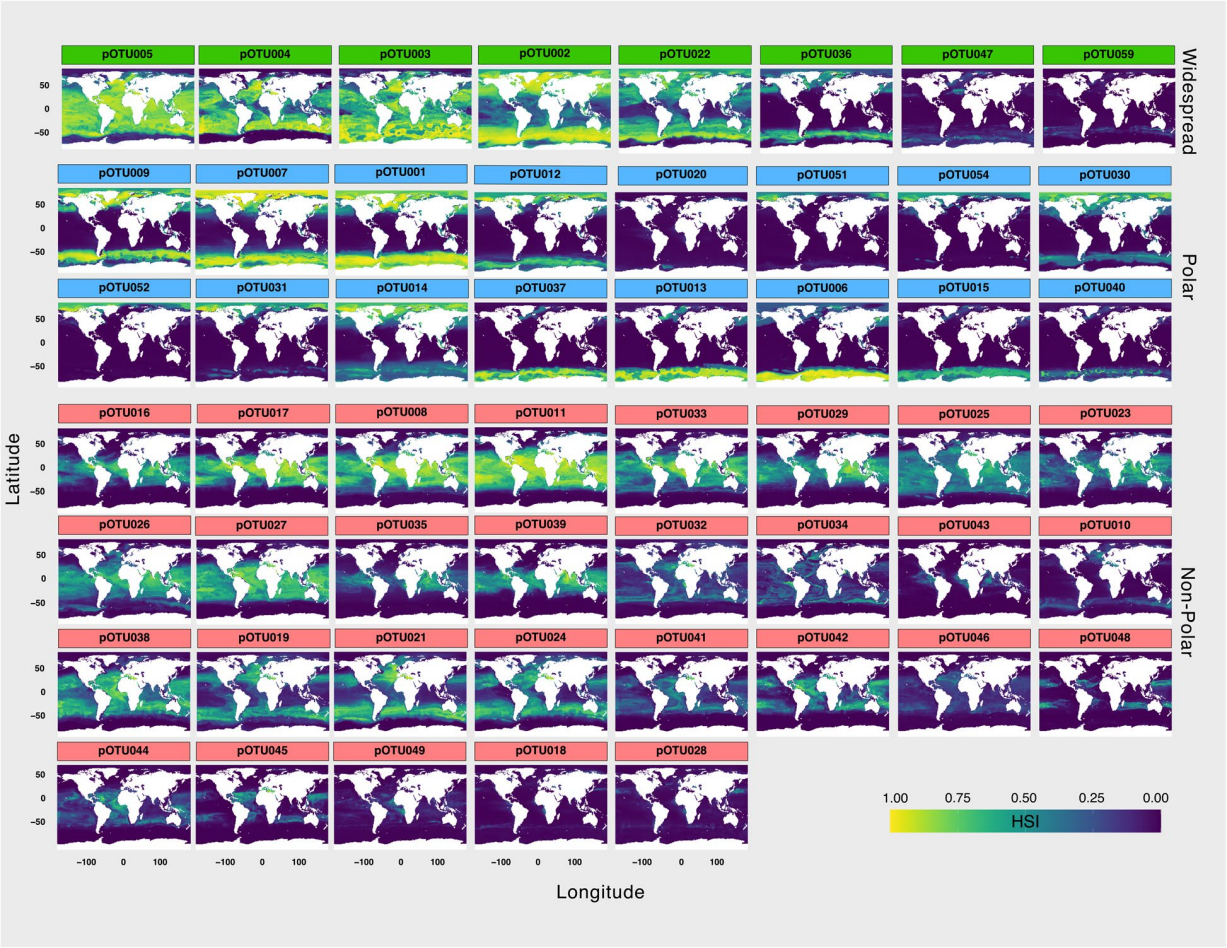
Latitudinal Distribution of Abundant pOTUs. This figure shows the habitat suitability index (HSI) of 53 abundant pOTUs for the global sunlit ocean, organized by their associated category based on the observed abundance and occupancy patterns (refer to Supplementary Fig. S6). Highest HSI index values indicate a higher probability of finding a pOTU in a given environment. Widespread pOTUs are indicated in green, Polar in blue, and Non-polar in red

Based on the observed abundance and occupancy pattern in the sunlit ocean, the pOTUs were classified into four categories: Low-abundant (LA), Widespread (W), Polar (P), and Non-polar (NP) (Supplementary Table S2). The LA category comprised pOTUs with a total abundance of less than 150 reads and occurrence in fewer than 50 samples. The majority of pOTUs (65 pOTUs) belonged to this category, but these contributed very little to total read abundance, less than 1%. The other three categories included fewer but more abundant pOTU (53 OTUs, total abundance > 150 reads, occurrence > 50 samples) and contributed similarly to the total read abundance (Supplementary Table S2). This classification was validated by calculating the statistically significant association of each pOTU to the oceans (IndVal.g; *p* < 0.05; Supplementary Table S2). The W category was represented by 8 pOTUs that were significantly associated with both polar and non-polar oceans (Fig. 5 and Supplementary Fig. S6). The P category consisted of 16 pOTUs that displayed significant association with the Arctic and/or the Southern Ocean and non-significant association with non-polar oceans (Fig. 5 and Supplementary Fig. S6). Lastly, the NP category showed a significant association only with non-polar oceans representing the largest group with the highest number of taxa (29 pOTUs).

Picozoa distributions predicted via SDMs provided further support for the classification of the abundant pOTUs into their respective category by revealing their distinct latitudinal distributions. The Widespread pOTUs displayed relatively high HSI index values across latitude ranges without a clear latitudinal pattern (Fig. 5 and Supplementary Fig. S6). Among P pOTUs, 7 had the highest HSI values in both polar regions, whereas 5 pOTUs tended to be highest in the Antarctic and for 4 pOTUs in the Arctic (Fig. 5 and Supplementary Fig. S6). NP pOTUs also showed different distribution patterns. Some of them exhibited high HSI values across the entire latitude range within non-polar limits, while others displayed a bimodal pattern, with the highest HSI values in the tropics decreasing in low latitudes near the equator (Fig. 5 and Supplementary Fig. S6).

### Ecological niche of Picozoa taxa

**Fig. 6:**
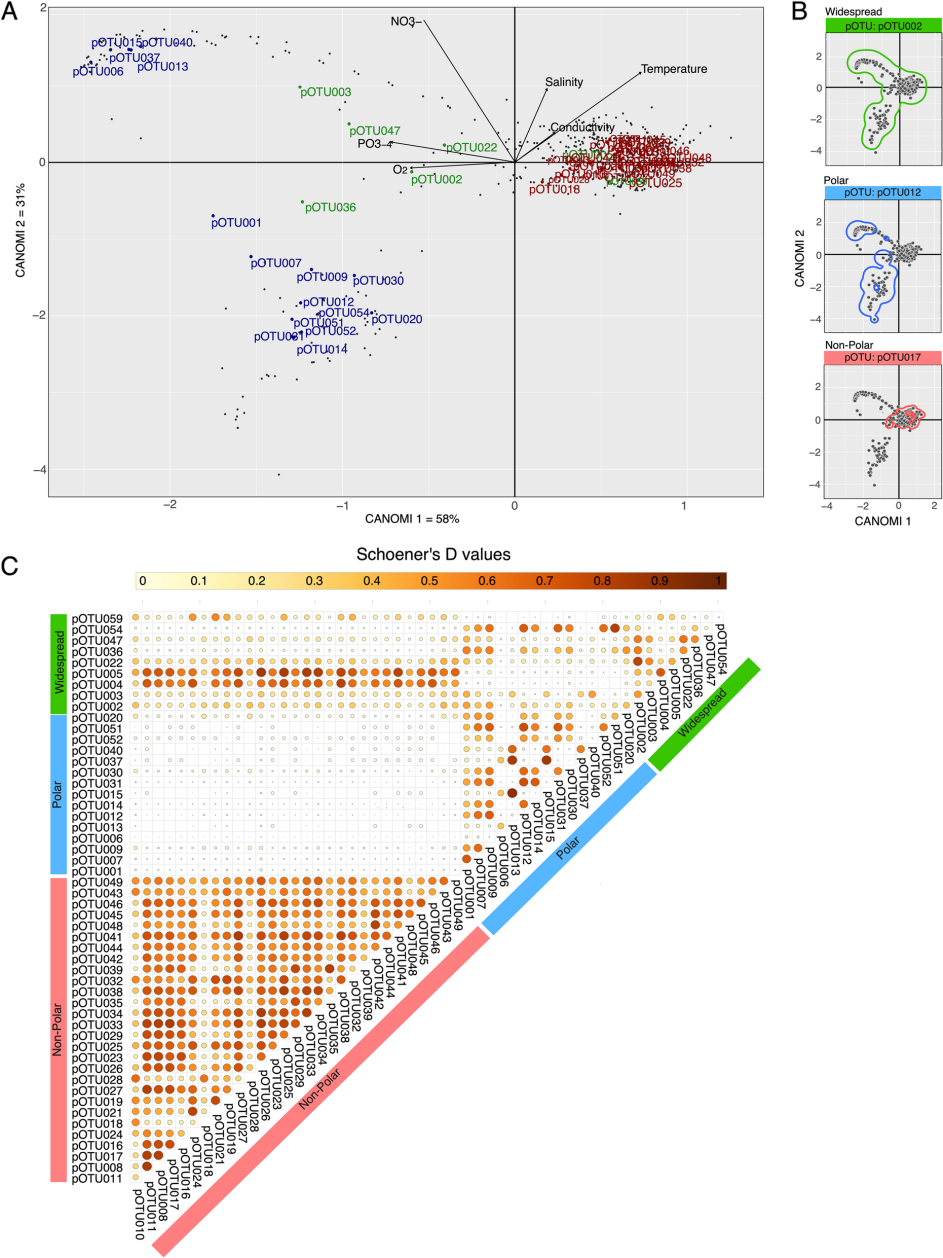
Ecological Niche and Niche Overlap of Abundant pOTUs. **A** Mean niche position of Polar (blue), Non-polar (red), and Widespread (green) pOTUs in the ecological space derived from the analyzed samples; grey dots represent the position of each sampling point in the ecological space. Arrowed lines depict canonical weightings of significant environmental factors contributing to the observed patterns. **B** Examples of estimated niche breadth using kernel density (colored lines represent 95% of probability distribution) in the environmental space for Widespread (green), Polar (blue), and Non-polar (red) pOTUs (refer to Supplementary Fig. S7); grey dots represent the position of each sampling point in the ecological space. **C** Schoener’s D index values representing niche overlap among pairs of abundant pOTUs. Highest Schoener’s D values indicate a higher niche overlap. Widespread pOTUs are indicated in green, Polar in blue, and Non-polar in red. Darker circles indicate higher overlap

We were further interested in exploring the realized environmental niche of each abundant pOTU. The CANOMI analysis revealed that pOTUs showing similar latitudinal patterns occupied similar positions in the ecological space (Fig. 6A). The analysis returned groups mainly partitioned by contrasting values of potential temperature, nitrates, phosphates, and oxygen concentrations (Fig. 6A). Salinity and conductivity showed a somewhat weaker contribution. Thus, the first CANOMI axis (eigenvalue = 1.55) explained about 58% of the total variance in the data, and it showed a strong positive correlation with temperature and a negative correlation with oxygen and phosphate concentrations. The CANOMI2 axis (eigenvalue = 0.83) explained about 31% of the total variance and was positively correlated with salinity and nitrates. The niches of Non-polar pOTUs were mostly associated with positive values of CANOMI1, corresponding to warmer waters with a lower concentration of nutrients (i.e., nitrates, phosphates). In contrast, Polar pOTUs showed a negative correlation with CANOMI1 and a broader distribution across CANOMI2 axis. Niches of all Polar pOTUs were associated with lower temperatures but segregated differently along the gradient of nitrate concentrations and salinity. Widespread pOTUs showed no clear segregation in the ecological space, although they seemed distributed mostly along gradients of oxygen and phosphate concentrations (Fig. 6A). Three Widespread pOTUs had niches closer to Non-polar taxa, whereas the remaining ones were associated with more temperate waters.

In the ecological space, we estimated the ecological niche breadth for each pOTU (see Fig. 6B for examples of pOTUs in the three categories). Overall, clear differences were observed among the three latitudinal groups, with members within each group displaying more ecological similarities than those from other latitudinal groups (Supplementary Fig. 7). As anticipated, Widespread pOTUs exhibited a broader estimated niche size compared to Polar and Non-polar pOTUs. However, a notable variability in the estimated niche breadth was also observed among pOTUs with similar latitudinal patterns, particularly for Polar and Widespread groups (Supplementary Fig. 7).

Using the estimated density data, we calculated niche overlap (Schoener’s *D* index) among pOTUs (Fig. 6C). The highest niche overlap was observed among Non-polar pOTUs. However, when comparing niche overlap values among Polar species, we found contrasting results. Higher overlap was observed for pOTUs distributed exclusively in the Arctic, Antarctic, or both polar regions. The distribution of Widespread pOTUs in the environment displayed diverse niche overlap patterns. Notably, pOTU004, pOTU005, and pOTU059 demonstrated a higher degree of overlap with Non-polar pOTUs, while pOTU036 exhibited overlap with Polar pOTUs. In contrast, the remaining pOTUs showed comparable levels of overlap with all pOTUs.

### Phylogenetic community structure and phylogenetic niche conservatism

We evaluated if pOTUs sharing the same ecological niche were also evolutionarily close. First, we used the MNTD index to assess the relatedness of pOTUs within communities. Interestingly, the analysis revealed that communities in high-latitude regions exhibited significantly higher MNTD values compared to those in medium and low latitudes (Kruskal–Wallis test, *p* < 0.05, Supplementary Table S4 and Supplementary Fig. S8). This finding suggests that pOTUs in polar communities are more distantly related than those in lower latitudes, indicating a potential link between evolutionary relatedness and ecological niche differentiation across latitudinal gradients.

**Fig. 7:**
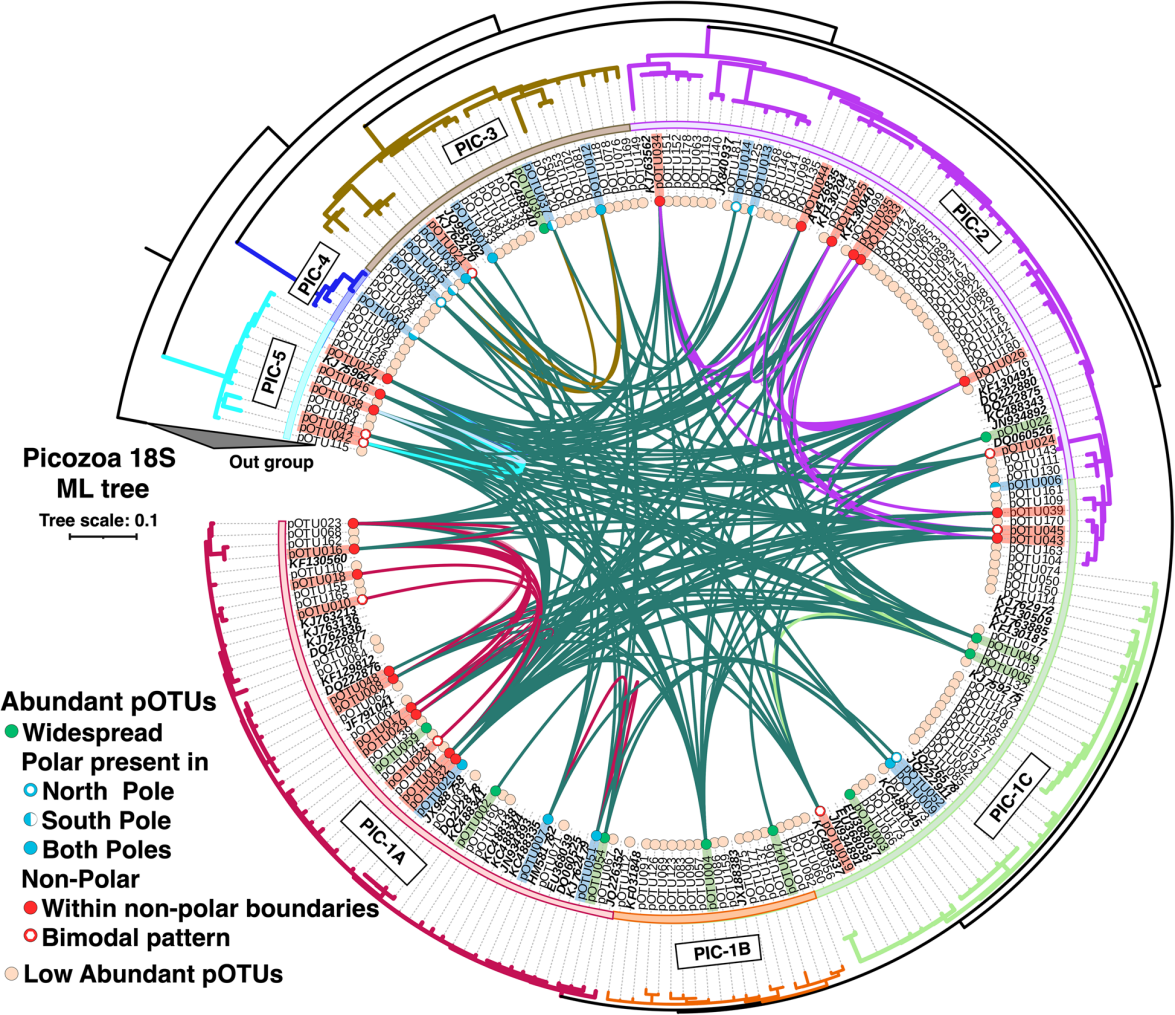
Phylogeny, Niche Similarity and Phylogenetic Overdispersion of Picozoa. 18S rRNA maximum likelihood circle tree (inverted) delineating Picozoa phylogenetic clades (refer to Fig. 3). The tree was constructed using reference sequences (highlighted in bold) and pOTUs considering 1000 replicates (refer to Fig. 3 and Supplementary Fig. S5). The colored circles inside indicate the category associated with each pOTU based on their abundance and occupancy pattern in the sunlit ocean (refer to Fig. 5 and Supplementary Fig. S6). Abundant pOTU are connected across the central circle by their niche overlap values, showing non-phylogenetic niche conservatism (only Schoener’s D values higher than 0.6 were considered, refer to Fig. 6). The colors of connecting lines indicate the niche overlap between pOTUs within the same clades (by corresponding color clades: strong pink for PIC-1A, soft green for PIC-1C, violet for PIC-2, greenish-brown for PIC-3, cyan for PIC-5) and from different clades (dark green)

Next, we examined phylogenetic niche conservatism. Analyzing the 18S rRNA tree, we observed a lack of clear correspondence between latitudinal patterns and phylogenetic relationships at the taxa level (Fig. 7). Additionally, pOTUs exhibiting high niche overlap were not segregated into a particular clade (Fig. 7). Notably, the correlation between phylogenetic distance among pOTUs and their niche overlap did not reveal any significant pattern (Supplementary Fig. S9a). To further investigate this relationship, we ran a Mantel correlogram analysis between pOTU environmental traits and phylogenetic distances which showed no significant correlation at any phylogenetic distance (Supplementary Fig. S9b).

## Discussion

Our results provide new insights into the diversity, distribution, ecological niches, and phylogenetic relationships of Picozoa, one of the most abundant microeukaryotic group in the global ocean. The phylum was represented by 179 Picozoa’s OTU, predominantly inhabiting marine environments. Phylogenetic analysis revealed that these pOTUs belonged to five distinct Picozoa clades. The assemblage structure showed a distinct latitudinal pattern, with polar assemblages showing a tendency to cluster separately from non-polar ones. Surprisingly, pOTUs occupying similar ecological niches were not closely related, suggesting a phylogenetic overdispersion within Picozoa. This could be attributed to competitive exclusion and the strong influence of the seasonal amplitude of variations in environmental factors, such as temperature, shaping physiological and ecological traits.

### What is the global distribution and diversity of Picozoa?

The presence of Picozoa has consistently accrued in molecular surveys since its initial discovery [7, 15, 19, 20, 25]. The EukBank database, targeting Eukaryotic communities across diverse types of environments, enables us to define Picozoa as a highly abundant, strictly marine group characterized by worldwide distribution in the ocean. Our findings align with previous surveys (e.g., in the Malaspina survey [21]) that have consistently observed Picozoa as one of the most abundant eukaryotic phyla despite its relatively low taxonomic diversity. The phylogenetic analysis of 18S rRNA genes from Picozoa sequences confirmed the existence of five distinct robust clades (PIC1-PIC5). This topology shows substantial congruence with previously published 18S rRNA gene trees, with PIC-1 corresponding roughly to BP1 plus BP3 (this being PIC-1C), PIC-2 to BP2 plus DB1, and PIC-3 to DB2 [14, 15]. Many of the deep branches shown in Schön et al. [13] were chimeras, except the two basal clades PIC-4 and PIC-5. Importantly, all of the pOTUs were clearly positioned within the established PIC clades, supporting the validity and representativeness of our Picozoa clades classification.

We observed a clear decline in diversity and abundance with depth, as reflected by distinct Shannon values between the surface and bathypelagic zones. This trend aligns with earlier observations in Picozoa and other heterotrophic lineages (e.g., Obiol et al. [21]). When examining assemblage compositions across global oceans, a clear latitudinal pattern emerges consistent with previous research highlighting variations in taxonomic composition within bacterial, archaeal, and protist communities in the Southern Ocean, Arctic Ocean, and non-polar oceans [77–83].

Recent studies have significantly advanced our understanding of the diversity and biogeography of specific protist groups, such as diatoms, green algae, and ciliates [84–87]. In particular, a recent study targeting the HF assemblage revealed clear biogeographic patterns in surface samples, with temperature and ocean basin identified as the primary factors influencing heterotrophic flagellates community variation [21]. Notably, the authors described Picozoa as one of the dominant groups in surface marine systems, with different taxa exhibiting varied distribution patterns, some displaying relatively constant abundances across samples, while others showing preferences for warmer or colder waters [21]. Here, expanding the geographic coverage of sampling, we observed similar distribution patterns. The classification of Picozoa into Widespread, Polar, and Non-polar groups unveiled distinct distribution strategies for different taxa within the phylum. Widespread taxa were found across various habitats, meaning they may be well-adapted to a wide range of environmental conditions, being not dependent on specific resources or interactions with other species. In contrast, Polar taxa demonstrated an affinity for cold polar environments, and Non-polar taxa were distributed only in warm and temperate waters. One surprising result was the distinct distribution patterns among Polar pOTUs, with some displaying high abundance in both polar regions, while others were exclusive to either the Arctic or Antarctic. These findings may provide evidence of endemic Picozoa taxa, indicating different evolutionary trajectories to thrive in polar conditions. However, dispersal limitation may substantiate the observed distribution patterns by the inability of pOTUs to colonize both poles due to physical or ecological barriers.

Variable levels of endemicity have been documented in microorganisms from the Antarctic and Arctic regions, including cyanobacteria, diatoms, and other bacterial and fungal species [77–83]. The unique and harsh environment of the polar regions, characterized by low temperatures, the lack of organic nutrients, liquid water, and high solar radiation, has led to the development of distinct microbial communities, with biodiversity being most prominent in the coastal regions, especially the Antarctic Peninsula [88]. However, as mentioned above, the effect of dispersal limitation may not be dismissed as an important factor influencing the observed distribution patterns, particularly in polar regions. Dispersal limitation refers to the inability of organisms to colonize new areas due to physical or ecological barriers [27]. The vast distance between the North and South polar regions results in geographical isolation, which limits the exchange of Picozoa between both polar regions. Besides, the ocean currents and environmental conditions as well as other biogeographic barriers such as continental landmasses and bathymetric features can also influence the dispersal of marine microbes between the North and South polar regions [89–91]. At this point, it is fair to consider that the observed distribution patterns could potentially be influenced also by technical limitations such as sequencing depth and the choice of clustering algorithms for defining pOTUs that might lead to an underrepresentation of certain taxa. Additionally, research campaigns targeting polar ecosystems frequently occur during the brief polar summers, potentially omitting crucial insights into the temporal variation of polar microbial communities. Previous studies have revealed that seasonality significantly influences Arctic marine life, including photoautotrophic organisms and bacterial communities. Factors like light availability, temperatures, water column properties, and sea ice coverage play substantial roles in molding the structure of microbial populations [22, 23, 92, 93]. Ignoring this intricate interplay could result in underestimating certain crucial components. For instance, specific OTUs might go undetected if their presence coincides solely with seasons outside the timeframe of current surveys.

### Does geographical distribution match ecological niches?

An intriguing question that follows the previous discussion is as follows: does the geographical distribution of pOTUs match their ecological niches? Abundant pOTUs were influenced by factors such as temperature, salinity, conductivity, oxygen, and nutrients, which determine their positions in the ecological space and, in general, aligned with latitudinal patterns. As anticipated, Widespread Picozoa demonstrates a broad ecological niche, indicating their ability to thrive across a diverse range of environmental conditions, similar to other protist groups. In contrast, Non-polar taxa tend to aggregate within a narrow environmental space, resulting in a comparatively high niche overlap. Polar OTUs segregate within the ecological space based on low-temperature conditions, with niche overlap varying between taxa, potentially influenced by specific adaptations to either the Arctic or Southern Oceans. Despite comparable climate drivers shaping the microbiome, the two polar oceans exhibit dissimilarities in salinity, water temperature, nutrient concentration, oceanic currents, and the influence of adjacent oceans [94–97]. These distinctive features in polar ecosystems likely propel the diversification of pOTUs functional traits, giving rise to a spectrum of ecological strategies finely tuned to exploit specific niches. Some studies have demonstrated that marine eukaryotic groups can exhibit differential distributions in the polar regions, with some species being more abundant in the Arctic and others in the Southern Ocean [77, 94, 95]. However, the differential taxa distributions within the same protist phylum, as presented in this work, are not usually reported in the literature. By employing an environmentally driven perspective, this study offers novel insights into divergent patterns of protist microbial diversity spanning from north to south.

It is important to acknowledge the limitations of the ecological niche analysis employed in this study. Our approach attempted to quantify the realized environmental niche of Picozoa by considering only abiotic environmental variables, such as temperature, salinity, nutrients, and oxygen. However, the niche of a species is influenced by a broader set of factors that determine its geographic distribution [98]. The realized niche encompasses three main classes of factors: (1) abiotic conditions, which impose physiological limits on a species’ ability to persist in an area; (2) biotic interactions, such as competition, predation, or mutualism, which further refine a species’ ability to maintain viable populations; and (3) dispersal and accessibility, which constrain a species’ ability to colonize suitable habitats. In the case of heterotrophic protists like Picozoa, biotic factors, particularly the availability and distribution of prey species, are likely to play a key role in shaping the realized niche. However, we were unable to include prey availability data in our niche models due to the lack of comprehensive information on the distribution and abundance of potential Picozoa prey organisms. The ecology of Picozoa, and in particular, their role in the microbial loop as heterotrophic organisms, is still poorly understood. By focusing solely on abiotic environmental variables, our niche modeling approach may have provided an incomplete characterization of the factors driving the geographic distribution of different Picozoa lineages. Future analyses incorporating biotic data, such as the distribution and abundance of prey organisms (e.g., bacteria, and small eukaryotes) into the species distribution modeling framework, would likely yield a deeper understanding of the underlying mechanisms driving the observed geographic distribution of Picozoa and that of other heterotrophic protists. Acknowledging these limitations is crucial for properly interpreting the conclusions drawn from the ecological niche analysis presented in this study. This is an important area for future research, as accounting for trophic relationships and community-level dynamics could improve the predictive power and ecological realism of distribution models of marine microbes.

### What are the eco-evolutionary processes that structure Picozoa communities?

The study of the relationship between the environmental preferences of Picozoa taxa and their phylogenetic community structure has revealed several intriguing insights on the eco-evolutionary processes that structure Picozoa communities. First, our assessment of the MNTD index shed light on the relatedness of pOTUs within communities. Communities in high latitudes displayed higher MNTD values, suggesting that pOTUs in polar communities are more distantly related compared to those in communities from medium and low latitudes [72]. This observation aligns with the results obtained from ecological niche analysis, reinforcing the concept of the distinctive ecological dynamics of polar ecosystems. Such environments frequently foster a diverse array of life forms that have adapted to thrive in challenging environmental conditions [96, 97]. Interestingly, the examination of the 18S rRNA phylogenetic tree revealed that pOTUs sharing a similar ecological niche were not closely related. The mantel test correlogram between pOTU environmental optima and pOTU phylogenetic distances confirmed this result. Furthermore, no clear correlation was observed when comparing the niche overlap between pOTUs with their phylogenetic distance [99]. Taken together, these results indicate that Picozoa communities exhibit phylogenetic overdispersion, a phenomenon that is opposite to phylogenetic niche conservatism. However, it cannot be ignored that the slow evolution rate of the 18S rRNA gene may not be sufficient to capture rapid changes between two operational taxonomic units (pOTUs), particularly in the context of phylogenetic niche conservatism. Despite this limitation, the 18S rRNA gene continues to serve as a valuable instrument for exploring evolutionary changes and biogeographic patterns. Thus, while many researchers consider PNC to be common, a review of case studies indicates that ecological and phylogenetic similarities are often not related. Consequently, ecologists should not assume that PNC exists but rather should empirically examine the extent to which it occurs. Considering the complexity involved, future efforts in studying Picozoa genomes will provide new insights to better understand the lack of PNC in Picozoa communities.

Several scenarios may explain why communities do not show PNC [38]. The first is competitive exclusion: if closely related species within a regional species pool are ecologically similar but there is a limit of resources, only distantly related species can coexist [100, 101]. The second is ecological divergence: closely related species might evolve different ecological traits to minimize resource overlap when living together [100, 101]. This divergence reduces ecological similarity among closely related species and diminishes or eliminates niche conservatism within a community [101]. The third is convergent adaptation: distantly related species might independently develop analogous traits or characteristics (like temperature tolerance) adapted to particular ecological features, despite having separate ancestral origins [101]. This adaptation can be repeated across many clades, resulting in distantly related species that are convergently adapted to the same ecological conditions. If only species with specific ecological attributes can coexist in a community, the community may exhibit phylogenetic overdispersion.

While the dataset used in this study does not permit a direct test of the mechanisms driving phylogenetic overdispersion, experimental evidence for heterotrophic protist species suggests that competition, particularly with phylogenetically related species, may lead to quicker exclusion, linked to phylogenetically conserved traits (e.g., mouth size [101]). Thus, it could be hypothesized that competitive exclusion plays an important role in driving a phylogenetic overdispersion in Picozoa assemblages. The observed patterns may also be linked to the influence of temperature, which plays a pivotal role in shaping physiological and ecological traits across various organizational levels. Temperature not only affects the behavior and performance of both predators and prey but also governs the ecological dynamics, ultimately molding the structure and function of ecological communities at diverse latitudes.

Expanding on recent studies, our work highlights the importance of understanding the species-level ecology and genomics of tiny ocean predators. The categorization of Picozoa into Widespread, Polar, and Non-polar groups unveils distinct distribution strategies for different taxa within the phylum, providing evidence of endemic Picozoa taxa with potentially different evolutionary histories adapted to polar conditions. The observed phylogenetic overdispersion challenges the concept of phylogenetic niche conservatism, indicating that closely related species do not necessarily share similar ecological niches. This deviation may be attributed to various factors, including competitive exclusion and the influence of temperature in shaping physiological and ecological traits across organizational levels. Thus, the hypothesis that drove our work was half fulfilled, since PNC could not be proven for Picozoa. However, it is important to highlight that technical biases of our dataset, previously discussed, could lead to misinterpretations of our results. Overall, this work contributes to advance our understanding of the evolutionary dynamics and ecological strategies employed by protists, underscoring the importance of future phylogenomic studies. The study highlights the need for continued research to unravel the mechanisms driving the observed patterns in protist communities.

## Supplementary Information

Additional file 1: Table S1. Metadata associated with samples from the EukBank dataset analyzed in this work. Table S2. List of picozoan operational taxonomic units (pOTUs) obtained from the EukBank amplicon dataset. Each row corresponds to one unique OTU identified based on 18S rDNA sequence. Table S3. PERMANOVA analysis to determine whether Picozoa communities structure differed among latitudinal rank. Table S4. Kruskal–Wallis analysis to determine whether pairwise NMTD values calculated from Picozoa communities differed among latitudinal rank. Fig. S1. Relative contribution of high-rank Protistan groups (indicated by different colors) to the total number of reads (represented by different areas) in the EukBank dataset (12,549 samples). Fig. S2. Picozoa presence across environments. Colors indicate the percentage of samples where Picozoa was detected in each environmental category. The grey bar indicates the percentage of samples where Picozoa was not detected. Fig. S3. Picozoa relative contribution (in yellow) to the total eukaryotic reads number (in white). Only samples where Picozoa constituted more than 5% of the total eukaryotic reads are presented. Fig. S4. Box Plot showing community features at the sunlit vs. dark ocean. For each community feature, the values were normalized to vary between 0 and 1. Significative differences are indicated with different letters (Test Student, p < s0.001). Fig. S5. 18S rDNA maximum likelihood phylogenetic tree based on Picozoa Reference Tree (Fig. 3) showing the phylogenetic relationships of the pOTUs from EukBank dataset. The tree was constructed with the GTRCATI considering 1000 replicate trees for topology and 1000 trees for bootstrapping using reference sequences and amplicon pOTUs. Fig. S6. Latitudinal Distribution of Abundant pOTUs. This figure shows the abundance patterns (log-transformed) of each abundant pOTU across latitudes, organized by their associated category based on abundance and occupancy patterns in the sunlit ocean. Widespread pOTUs are indicated in green, Polar in blue, and Non-polar in red. Fig. S7. Estimated niche breadth using kernel density for Widespread (green), Polar (blue), and Non-Polar (red) pOTUs. Fig. S8. MNTD values by latitudinal rank for Picozoa Communities (see Supplementary Table S4 for pairwise comparison statistical test). Fig. S9. (a) Relationships between niche overlap (Schoener's D index) and phylogenetic distance (normalized to vary between 0 and 1) for abundant pOTUs, confirming the absence of niche conservatisms in Picozoa. Specific color dots highlight pairwise relationships among pOTUs within the same clade, while grey dots represent pairwise relationships between pOTUs from different clades. (b) Mantel correlograms (Pearson correlations) between pOTU environmental optimal distances and phylogenetic distances with 9 999 permutations. Significant correlations (*P* < 0.05) were not detected over phylogenetic distances. For each phylogenetic distance, bin phylogenetic distances were normalized to vary between 0 and 1 before analysis.

## Data Availability

The data, metadata, and scripts used for the analysis are available on the GitHub repository: https://github.com/hubermp/picozoa_distribution. Raw sequence data supporting this study's findings are deposited in the European Nucleotide Archive and the accession numbers are provided in the Supplementary Table S1.
