# Supplementary material for: Global distribution, diversity, and ecological niche of Picozoa, a widespread and enigmatic marine protist lineage
Source: Microbiome. 2024 Sep 4;12:162. doi: 10.1186/s40168-024-01874-1 (PMC11373171; doi:10.1186/s40168-024-01874-1)

**Supplementary Information for**

**Global Distribution, Diversity and Ecological Niche of Picozoa, a widespread and enigmatic marine protist lineage**

Paula Huber^1*^, Daniele De Angelis^2^, Hugo Sarmento^1*^, Sebastián Metz^3^, Caterina R. Giner^4^, Colomban De Vargas^5,6^, Luigi Maiorano^2^, Ramon Massana^4^, Ramiro Logares^4*^

**This file includes:**

Figs. S1 to S9

**Corresponding author:**

Huber, Paula

Universidade Federal de São Carlos

Departamento de Hidrobiologia

Rodovia Washington Luís, Km 235 - Caixa Postal 676

CEP 13565-905 - São Carlos (SP) Brasil

E-mail: mariapaulahuber@gmail.com

**Supplementary Fig. S1:** Relative contribution of high-rank Protistan groups (indicated by different colors) to the total number of reads (represented by different areas) in the EukBank dataset (12,549 samples).


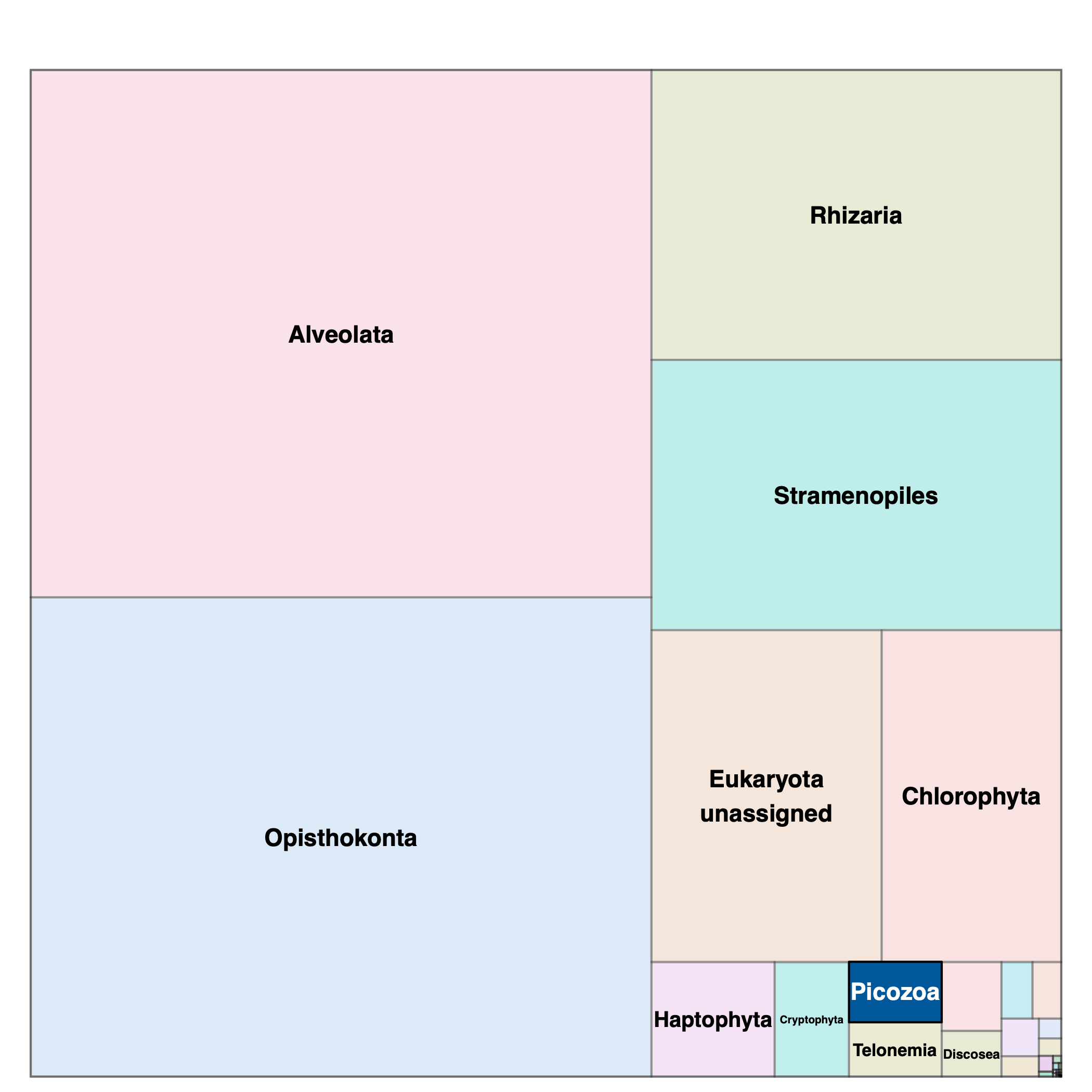


**Supplementary Fig. S2:** Picozoa presence across environments. Colors indicate the percentage of samples where Picozoa was detected in each environmental category. The grey bar indicates the percentage of samples where Picozoa was not detected.


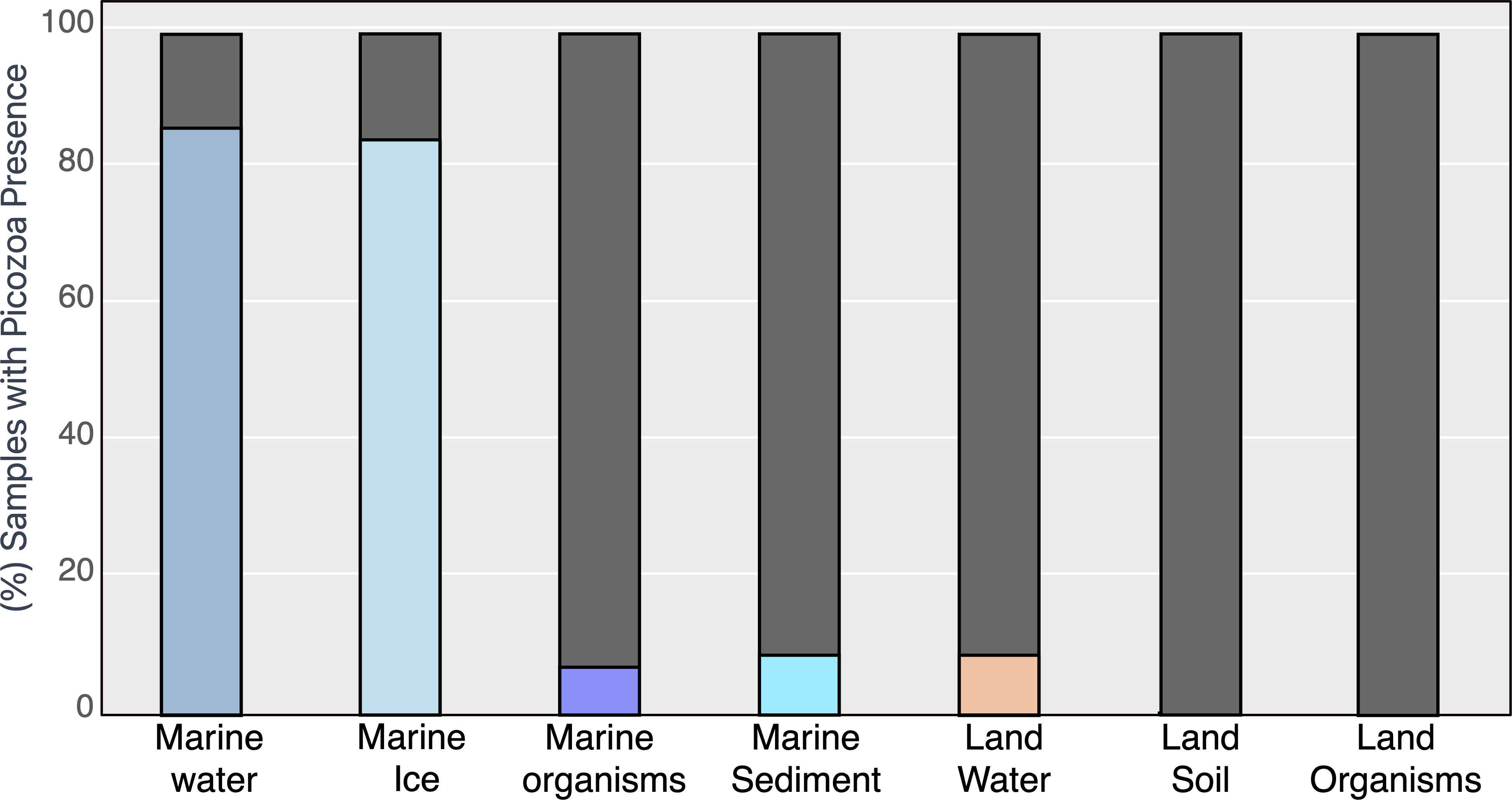


**Supplementary Fig. S3**: Picozoa relative contribution (in yellow) to the total eukaryotic reads number (in white). Only samples where Picozoa constituted more than 5% of the total eukaryotic reads are presented.


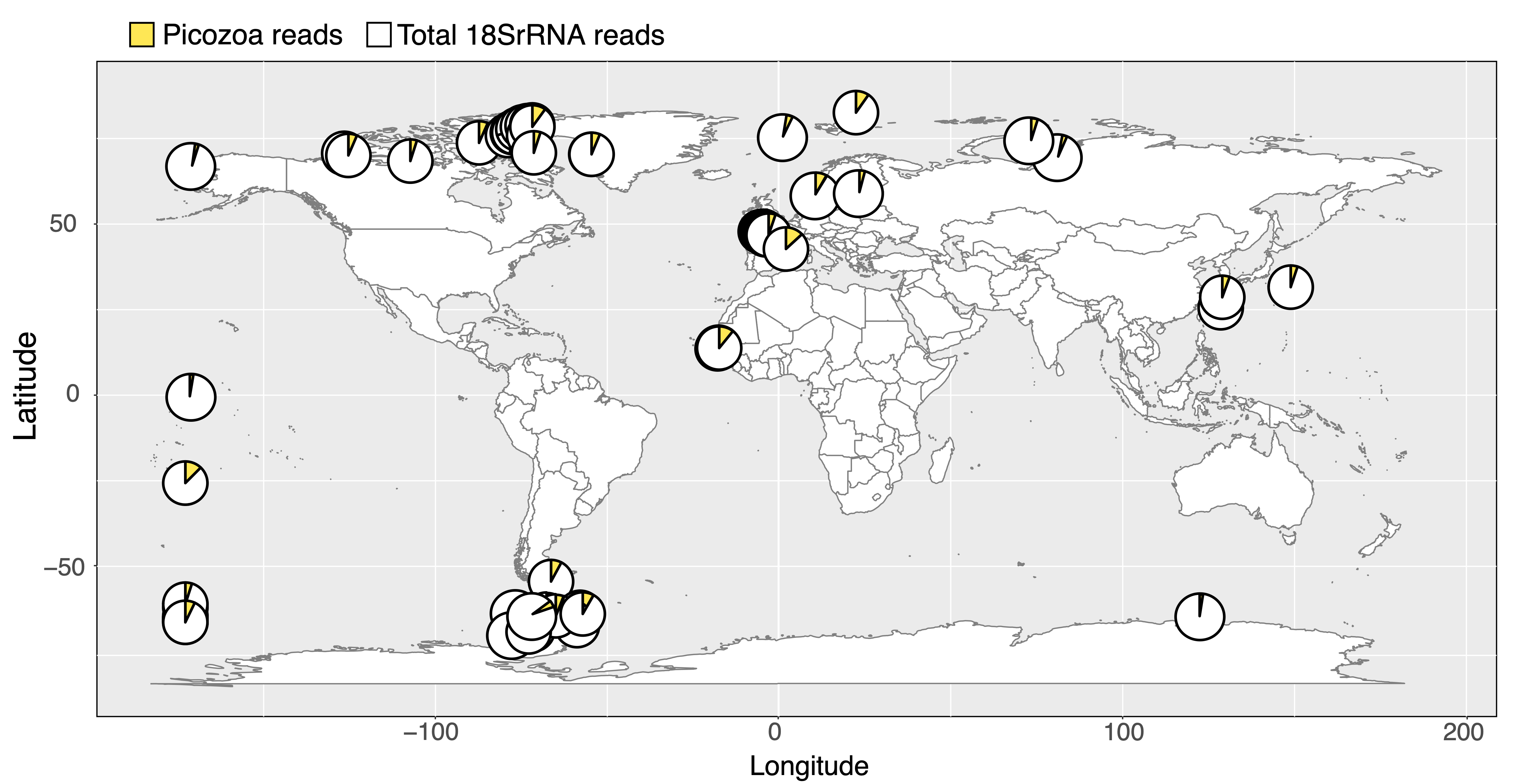


**Supplementary Fig. S4:** Box Plot showing community features at the sunlit vs. dark ocean. For each community feature, the values were normalized to vary between 0 and 1. Significative differences are indicated with different letters (Test Student, p<s0.001)

**Supplementary Fig. S5:** 18S rDNA maximum likelihood phylogenetic tree based on Picozoa Reference Tree (Fig. 3) showing the phylogenetic relationships of the pOTUs from EukBank dataset. The tree was constructed with the GTRCATI considering 1 000 replicate trees for topology and 1000 trees for bootstrapping using reference sequences and amplicon pOTUs.


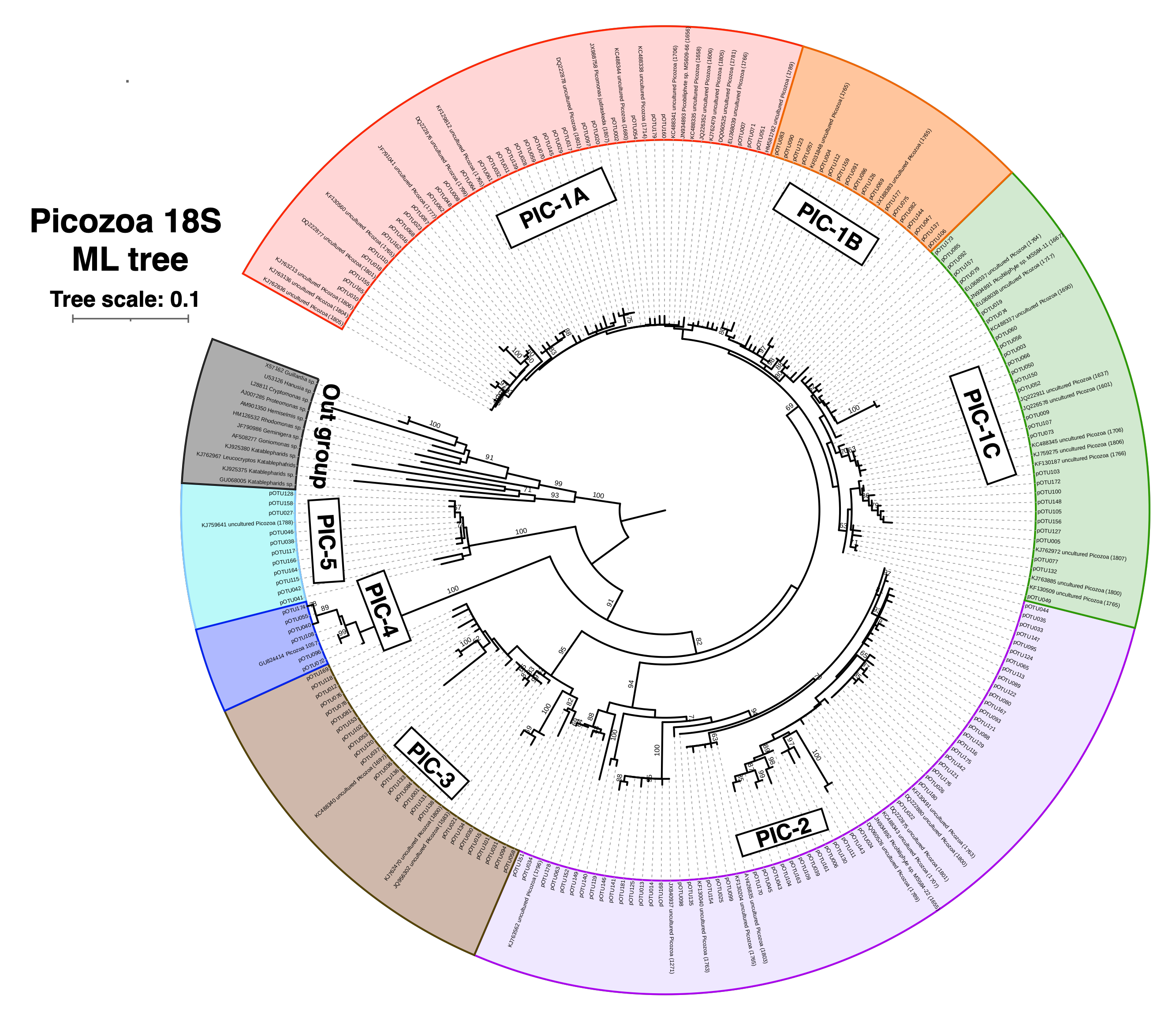


**Supplementary Fig. S6:** **Latitudinal Distribution of Abundant pOTUs.** This figure shows the abundance patterns (log-transformed) of each abundant pOTU across latitudes, organized by their associated category based on abundance and occupancy patterns in the sunlit ocean. Widespread pOTUs are indicated in green, Polar in blue, and Non-polar in red.

**
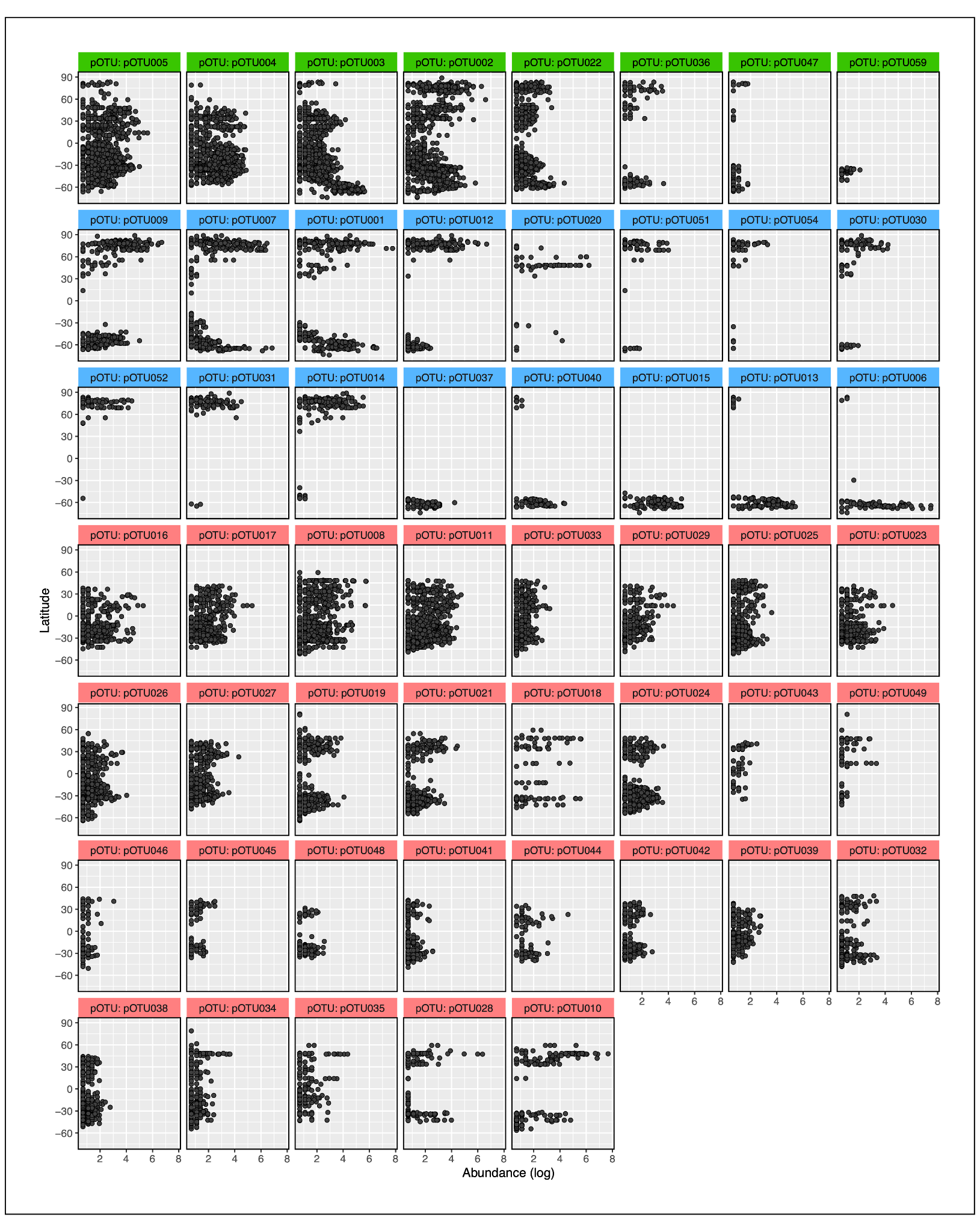
**

**Supplementary Fig. S7:** Estimated niche breadth using kernel density for Widespread (green), Polar (blue), and Non-Polar (red) pOTUs.

**
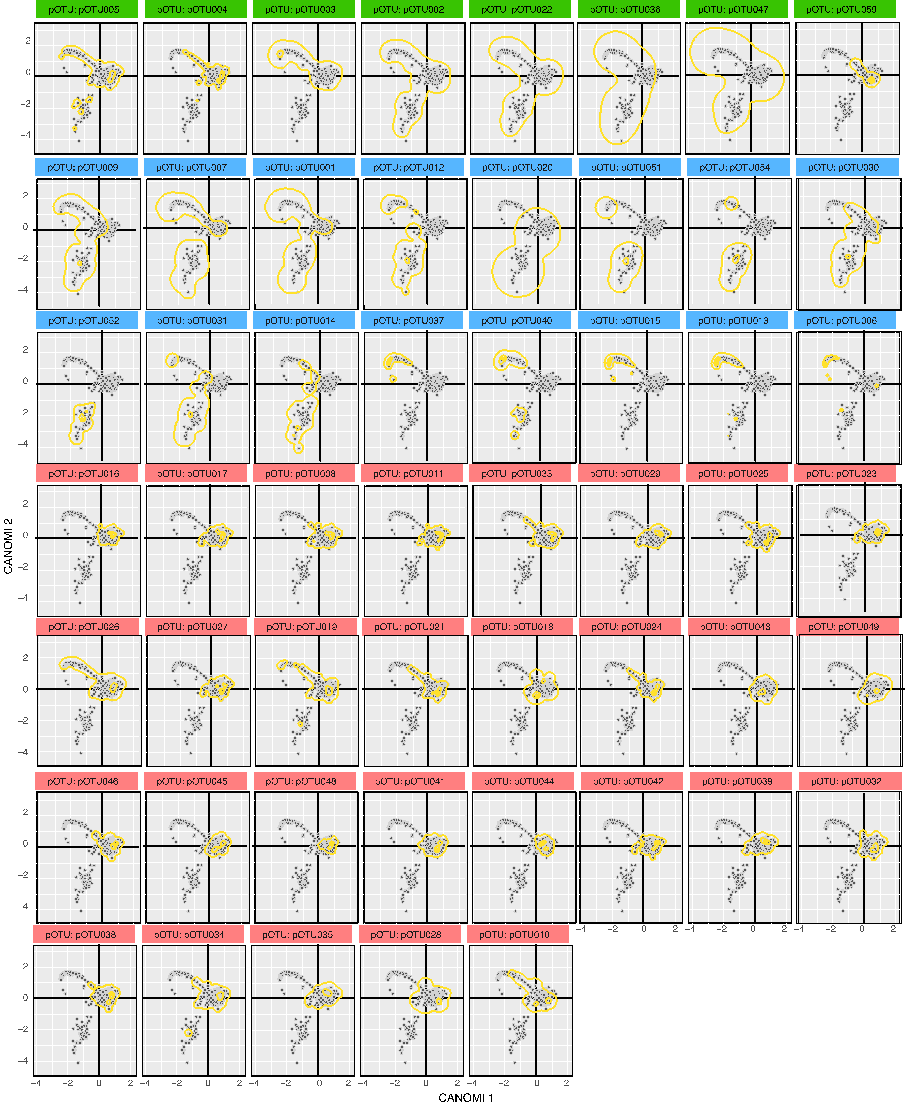
**

**Supplementary Fig. S8:** MNTD values by latitudinal rank for Picozoa Communities (see Supplementary Table S4 for pairwise comparison statistical test).


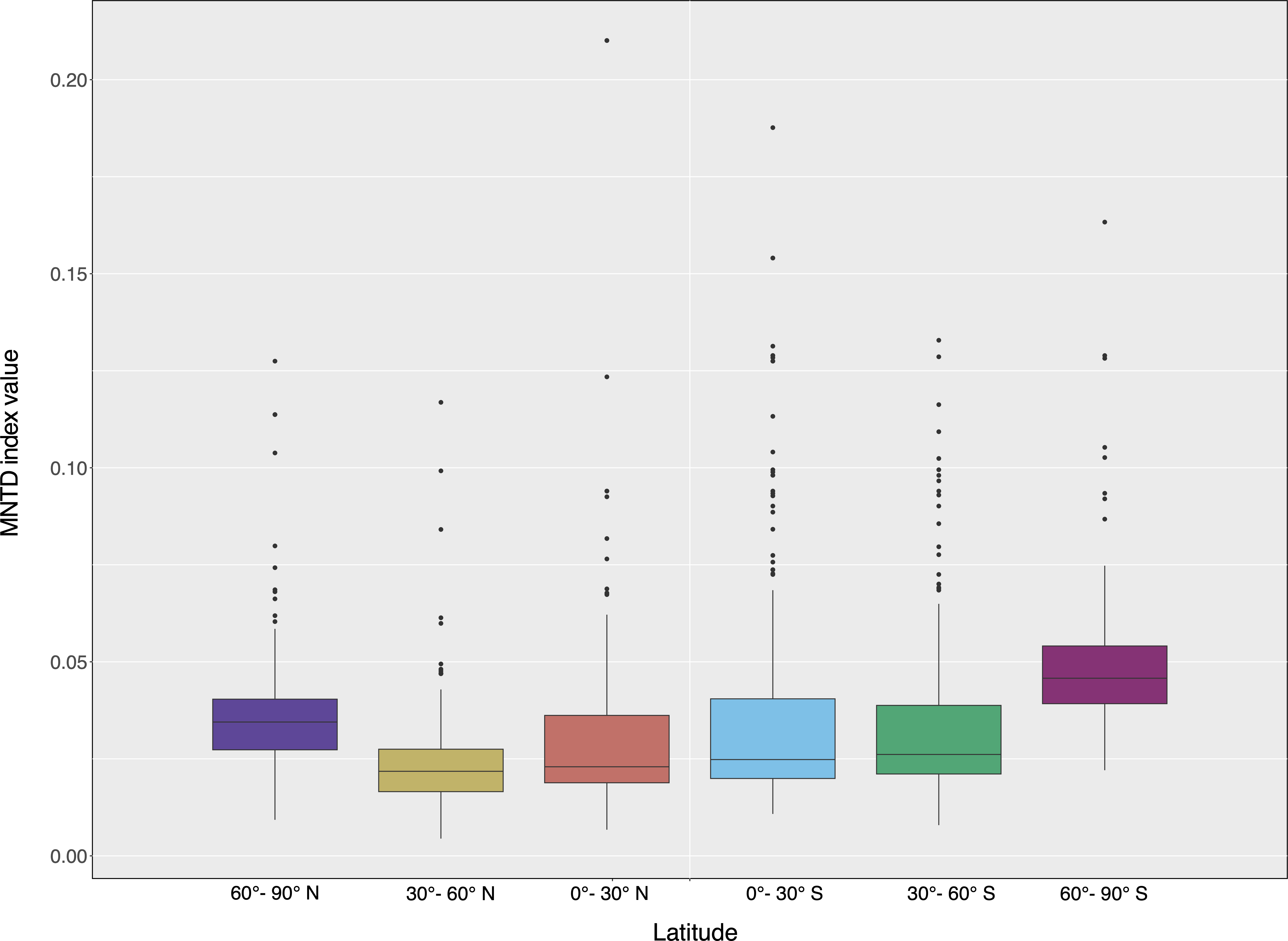


**Supplementary Fig. S9:** (a) Relationships between niche overlap (Schoener's D index) and phylogenetic distance (normalized to vary between 0 and 1) for abundant pOTUs, confirming the absence of niche conservatisms in Picozoa. Specific color dots highlight pairwise relationships among pOTUs within the same clade, while grey dots represent pairwise relationships between pOTUs from different clades. (b) Mantel correlograms (Pearson correlations) between pOTU environmental optimal distances and phylogenetic distances with 9 999 permutations. Significant correlations (P<0.05) were not detected over phylogenetic distances. For each phylogenetic distance, bin phylogenetic distances were normalized to vary between 0 and 1 before analysis.


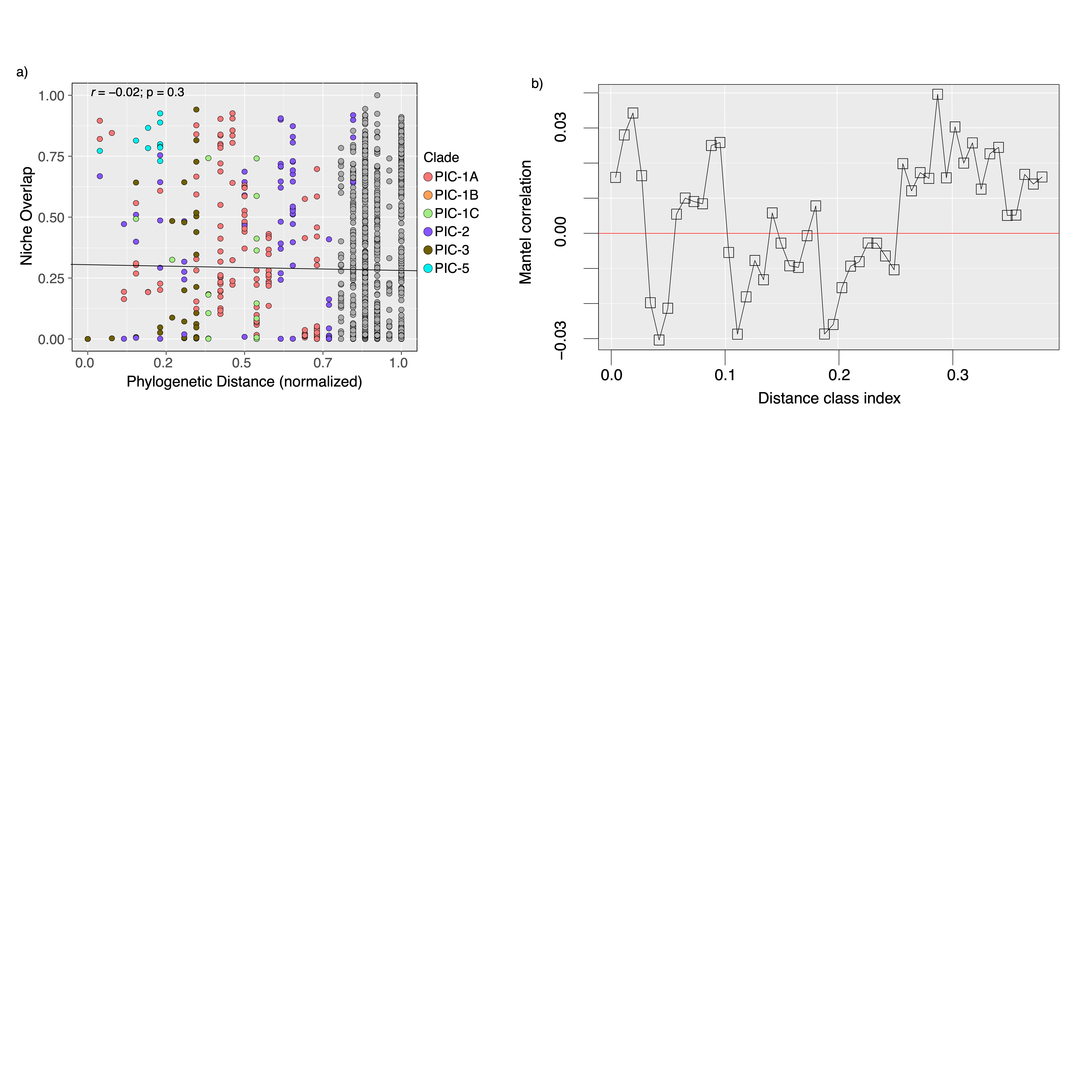

Supplement: Supplementary file 2 — Additional file 1: Table S1. Metadata associated with samples from the EukBank dataset analyzed in this work. Table S2. List of picozoan operational taxonomic units (pOTUs) obtained from the EukBank amplicon dataset. Each row corresponds to one unique OTU identified based on 18S rDNA sequence. Table S3. PERMANOVA analysis to determine whether Picozoa communities structure differed among latitudinal rank. Table S4. Kruskal–Wallis analysis to determine whether pairwise NMTD values calculated from Picozoa communities differed among latitudinal rank. Fig. S1. Relative contribution of high-rank Protistan groups (indicated by different colors) to the total number of reads (represented by different areas) in the EukBank dataset (12,549 samples). Fig. S2. Picozoa presence across environments. Colors indicate the percentage of samples where Picozoa was detected in each environmental category. The grey bar indicates the percentage of samples where Picozoa was not detected. Fig. S3. Picozoa relative contribution (in yellow) to the total eukaryotic reads number (in white). Only samples where Picozoa constituted more than 5% of the total eukaryotic reads are presented. Fig. S4. Box Plot showing community features at the sunlit vs. dark ocean. For each community feature, the values were normalized to vary between 0 and 1. Significative differences are indicated with different letters (Test Student, p < s0.001). Fig. S5. 18S rDNA maximum likelihood phylogenetic tree based on Picozoa Reference Tree (Fig. 3) showing the phylogenetic relationships of the pOTUs from EukBank dataset. The tree was constructed with the GTRCATI considering 1000 replicate trees for topology and 1000 trees for bootstrapping using reference sequences and amplicon pOTUs. Fig. S6. Latitudinal Distribution of Abundant pOTUs. This figure shows the abundance patterns (log-transformed) of each abundant pOTU across latitudes, organized by their associated category based on abundance and occupancy patterns in the su [file 40168_2024_1874_MOESM1_ESM.zip › Huber_etal_Picozoa_SupplementaryInformation_RW_ESM.docx]
